# An asymmetric allelic interaction drives allele transmission bias in interspecific rice hybrids

**DOI:** 10.1038/s41467-019-10488-3

**Published:** 2019-06-07

**Authors:** Yongyao Xie, Jintao Tang, Xianrong Xie, Xiaojuan Li, Jianle Huang, Yue Fei, Jingluan Han, Shuifu Chen, Huiwu Tang, Xiucai Zhao, Dayun Tao, Peng Xu, Yao-Guang Liu, Letian Chen

**Affiliations:** 1 State Key Laboratory for Conservation and Utilization of Subtropical Agro-Bioresources, South China Agricultural University, Guangzhou, 510642 China; 2 Guangdong Provincial Key Laboratory of Protein Function and Regulation in Agricultural Organisms, South China Agricultural University, Guangzhou, 510642 China; 3 Key Laboratory of Plant Functional Genomics and Biotechnology of Guangdong Provincial Higher Education Institutions, South China Agricultural University, Guangzhou, 510642 China; 4 College of Life Sciences, South China Agricultural University, Guangzhou, 510642 China; 5 Food Crops Research Institute, Yunnan Academy of Agricultural Sciences, Kunming, 650200 China; 6 Present Address: Xishuangbanna Tropical Botanical Garden, Chinese Academy of Sciences, Kunming, 650200 China

**Keywords:** Agricultural genetics, Development, Plant evolution, Fertilization

## Abstract

Hybrid sterility (HS) between *Oryza sativa* (Asian rice) and *O*. *glaberrima* (African rice) is mainly controlled by the *S1* locus. However, our limited understanding of the HS mechanism hampers utilization of the strong interspecific heterosis. Here, we show that three closely linked genes (*S1A4*, *S1TPR*, and *S1A6*) in the African *S1* allele (*S1-g*) constitute a killer-protector system that eliminates gametes carrying the Asian allele (*S1-s*). In Asian–African rice hybrids (*S1-gS1-s*), the S1TPR-S1A4-S1A6 interaction in sporophytic tissues generates an abortion signal to male and female gametes. However, S1TPR can rescue *S1-g* gametes, while the *S1-s* gametes selectively abort for lacking S1TPR. Knockout of any of the *S1-g* genes eliminates the HS. Evolutionary analysis suggests that *S1* may have arisen from newly evolved genes, multi-step recombination, and nucleotide variations. Our findings will help to overcome the interspecific reproductive barrier and use Asian–African hybrids for increasing rice production.

## Abstract (web summary)

Our limited understanding of the hybrid sterility (HS) mechanism in Asian–African rice hybrids hampers utilization of the interspecific heterosis for rice production. Here, the authors identify *S1*-mediated HS-related tripartite gamete killer-protector system, and explore their evolutionary relationship.

## Introduction

Hybrid sterility (HS) is a major mechanism of postzygotic reproductive isolation, which can limit reproduction among divergent populations during speciation and thus contribute to the maintenance of species identity^1,2^. The classic Bateson–Dobzhansk–Muller (BDM) model^3^ attributes postzygotic reproductive isolation to an incompatible genetic interaction between divergent alleles of at least two loci^4,5^, although mounting evidence suggests that HS caused by the interaction of alleles comprising multiple genes at a single locus also fits the BDM model^6–15^. The HS loci act as selfish genetic elements, which can eliminate the gametes carrying competing alleles, thus gaining a transmission advantage over other alleles, similar to meiotic drive. This can result in distorted segregation and non-Mendelian transmission of the alleles in the progenies^6–15^. The killer–protector system and killer meiotic driver models have been proposed to explain the biased allele transmission of HS in eukaryotes^14,16^. In this model, the killer gene (usually functioning in the sporophyte) produces a detrimental sterility signal that indiscriminately kills all meiotic cells^16^. The protector gene (usually tightly linked to the killer gene) functions in the gamete to eliminate this detrimental effect and rescue the gamete that contains the correct allele of the protector gene. Therefore, gametes carrying the protector gene survive; gametes lacking the protector gene die^16^.

The *Oryza* genus comprises 21 wild species and 2 cultivated species, Asian rice (*O*. *sativa* L., including the subspecies *japonica* and *indica*) and African rice (*O*. *glaberrima* Steud.)^17,18^. The broad genetic diversity within *Oryza* can contribute to heterosis, also known as hybrid vigor, in which the hybrid performs better than its parental inbred lines^19,20^. However, HS of different forms (e.g., male sterility, female sterility, and both male and female sterilities) is common between different species and subspecies in the *Oryza* genus and this HS hinders the ability of plant breeders to use the strong heterosis in the production of high yield, robust hybrid rice. Therefore, understanding the mechanisms of the HS could enable breeding of improved hybrid rice. A number of HS loci have been genetically studied in *Oryza*^19,20^ and several HS genes have been cloned from various loci, including the single-locus HS loci *Sa*, *S5*, *HSA1*, *S7*, *S1*, *Sc*, *qSHMS7*, *ESA1*, and the two-locus *DPL1*/*DPL2*, *S27*/*S28*, and *DGS1*/*DGS2* pairs^8,12,14,15,21–29^. Despite extensive studies of these loci, our understanding of the molecular mechanisms governing HS and its effect on genome evolution remains limited.

*S1* is a typical single-locus-type HS locus that affects hybrids produced by crossing African and Asian rice^23,27,30–32^. In these Asian–African rice hybrids, male and female gametes carrying the Asian rice *S1* allele (*S1-s*) are selectively aborted, leading to very strong preferential transmission of the African rice *S1* allele (*S1-g*), which reaches an allele frequency of ca. 0.95 in F_2_ progeny^27^. Recently, we cloned *OgTPR1*, the peptidase-encoding causal gene of the HS effect of the *S1* locus; another *S1*-related gene, *SSP*, also encoding a peptidase, was later identified by Koide and colleagues^23^. Despite these advances, the molecular mechanism governing the selective abortion and survival of gametes with different alleles in interspecific and intersubspecific hybrids in plants remains poorly understood, and the evolutionary origins of HS loci and their relationship to speciation remain largely unknown. Here, we identify additional HS-related genes at *S1*, describe the *S1* tripartite gamete killer–protector system, and explore the evolutionary relationship of this complex locus with the allopatric speciation of the related *Oryza* species.

## Results

### The *S1A4* gene is required for *S1* HS

**Fig. 1:**
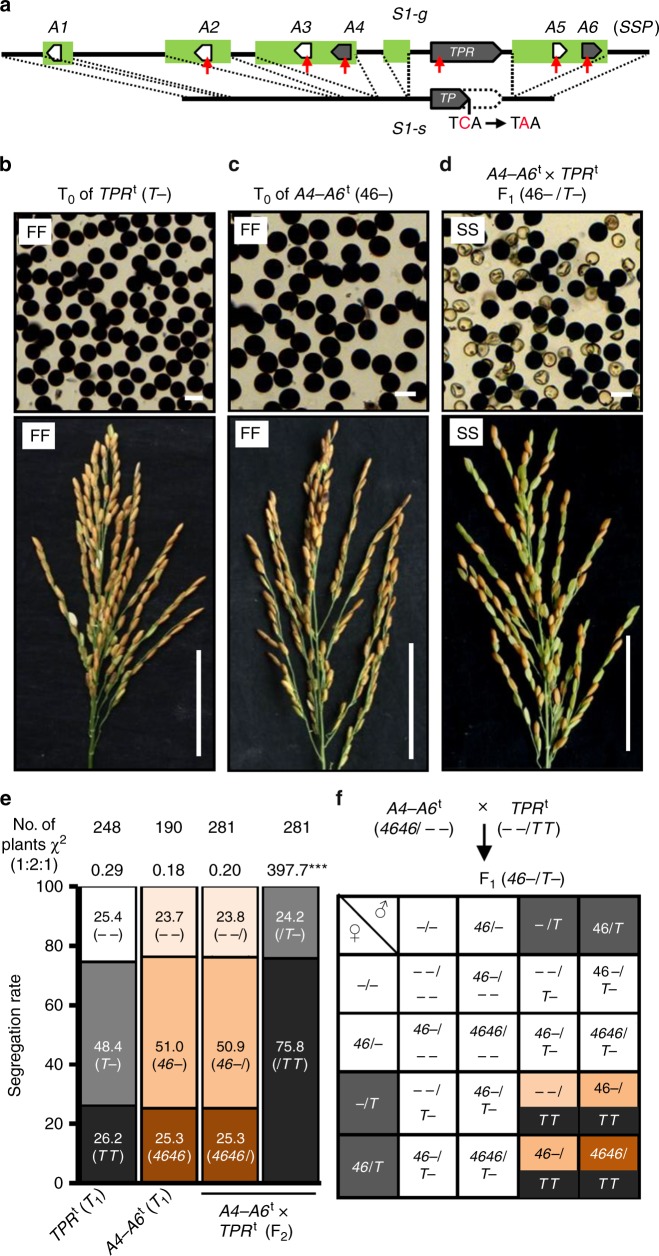
*S1A4*-*S1TPR*-*S1A6* constitutes a killer–protector system. **a** The structures of the African rice allele *S1-g* (*g*) and Asian rice allele *S1-s* (*s*). A point (C-to-A) mutation of *S1TPR* (*TPR*) results in a premature stop codon in *S1TP* (*TP*). African rice-specific sequences are in green. Six putative genes specific to African rice, *S1A1*–*S1A6* (*A1*–*A6*), were located in *S1-g*. Red arrows, CRISPR/Cas9 target sites. **b**–**d** The fertility of the transgenic plants of RP-*s* (the recurrent parent of *O*. *sativa* ssp. *japonica* with *ss*) and their hybrids carrying different combinations of the transgenes *TPR*^t^ (*T*) and linked *A4*–*A6*^t^ (*46*). The T_0_ plants carrying one (**b**) or two (**c**) transgenes were fully fertile (FF, ~95% fertility), as observed in their pollen (top) and spikelets (bottom); however, the co-existence of the three transgenes (all in hemizygous state; ‘–’ denotes absence of the T-DNA/transgene in the chromosome site) induced semi-sterility (SS, 45~55% sterility) of the pollen and spikelets (green spikelets are sterile). **d** Bars, 50 μm for pollen and 5 cm for panicles. **e** The segregation of the *A4–A6*^t^ and *TPR*^t^ transgenes in the T_1_ family, and the segregation of the *A4–A6*^t^ in F_2_ population derived from *A4*–*A6*^t^ (homozygote) × *TPR*^t^ (homozygote) fit the 1:2:1 ratio, but the segregation of the *TPR*^t^ transgene in this F_2_ population was significantly distorted (***P < 0.001 in the *Χ*^2^ test) from the ratio. **f** A deduced model for the segregation behavior of the transgenes in the F_2_ progeny derived from *A4*–*A6*^t^ × *TPR*^t^. The F_1_ male and female gametes containing *T* are fertile, and those lacking *T* are generally sterile. The color codes are consistent with the genotype of the individuals in the F_2_ population. Black represents homozygous *TPR*^t^; dark orange represents homozygous *A4*–*A6*^t^; orange represents hemizygous *A4*–*A6*^t^; light orange represents lacking *A4*–*A6*^t^. Source data of (**e**) are provided as a Source Data file

Our previous study revealed the existence of structural variation between the African rice *S1* allele (*S1-g*) and the Asian rice *S1* allele (*S1-s*)^27^. We found that *OgTPR1* (hereafter named *S1TPR*) at *S1-g* is required for *S1*-mediated HS. Moreover, *S1-s* contains an *S1TPR* allele, named *S1TP*, which is a truncated form of *S1TPR* due to a premature stop codon caused by a single-nucleotide mutation (Fig. 1a)^27^. According to our sequence analysis, besides *S1TPR*, the *S1-g* region contains six African rice-specific putative genes, *S1A1*–*S1A6* (Fig. 1a). We determined the expression profiles of these genes in a near-isogenic line carrying *S1-g* (NIL-*g*) and its recurrent parental line RP-*s* (*japonica* rice carrying *S1-s*), and their F_1_ plants. This showed that *S1A2–S1A6* were transcriptionally active in anthers and young panicles (Supplementary Figs. 1a and 2). Moreover, *S1TPR* was expressed at high levels in the microspores (Supplementary Fig. 1b)

To test whether *S1TPR* is sufficient to cause gamete abortion, we generated transgenic plants that contained an *S1TPR* transgene (*S1TPR*^t^) in the RP-*s* background. The T_0_ plants hemizygous for *S1TPR*^t^ (*S1TPR*^t^–, where dash indicates absence of the T-DNA/transgene in the chromosome site) had fully fertile pollen and spikelets (Fig. 1b), indicating that *S1TPR* is not sufficient to induce HS. These results suggest that *S1* HS requires other *O*. *glaberrima*-specific *S1* component(s) in addition to *S1TPR*.

To investigate whether any of the anther- and panicle-expressed *S1* genes (*S1A2*–*S1A6*) are involved in *S1* HS, we used CRISPR/Cas9 to individually knock out their functions in NIL-*g*. All the obtained knockout mutants (*s1a2–s1a6*) showed normal male and female fertility, which indicates that these genes are not essential for gamete development (Supplementary Fig. 3a and Supplementary Table 1). If a specific gene is required for *S1* HS, knocking it out in NIL-*g* should create a neutral allele that does not show HS in crosses with RP-*s*. When these mutants were crossed with RP-*s*, the mutant F_1_ plants containing the *s1a2*, *s1a3*, or *s1a5* mutant alleles were semi-sterile (ca. 50% sterile pollen grains and spikelets) (Supplementary Fig. 3b and Supplementary Table 2), like the RP-*s* × NIL-*g* F_1_ plants (see below Fig. 3a). However, the mutant F_1_ plants containing the *s1a4* and *s1a6* alleles were fully fertile (Supplementary Fig. 3b and Supplementary Table 2), suggesting that *S1* HS requires *S1A4* and *S1A6*. This result is consistent with a recent report that *SSP*, which corresponds to *S1A6*, is involved in *S1* HS^23^. Moreover, we found that in the *s1a4* and *s1a6* mutant lines, agronomic traits including plant architecture, grain length, grain width, plant height, panicle length, and grain number per panicle were not significantly different compared to NIL-*g*, but their 1000-grain weight was slightly heavier than that of NIL-*g* (Supplementary Fig. 4). These results suggest that *S1A4* and *S1A6* may have a pleiotropic effect on the seed-filling process in addition to their roles in HS.

### *S1A4*-*S1TPR*-*S1A6* constitutes a killer–protector system

To understand whether *S1A4*, *S1TPR*, and *S1A6* are sufficient to kill gametes, we transformed RP-*s* with transgenes containing different combinations of these three genes, then looked for sterility and distortion of segregation of the transgenes. If the killing process requires all three genes, gamete abortion would not occur in plants carrying only one or two of these components. Indeed, all the plants transformed with one (*S1TPR*^t^, *S1A4*^t^, or *S1A6*^t^), or two transgenes (the *S1A4–S1A6*^t^ and *S1TPR-S1A6*^t^ transgenes carrying two genes, or the pyramided transgenes in the F_1_ of *S1A4*^t^ × *S1TPR*^t^), all in a hemizygous state, did not show the typical semi-sterile phenotypes of pollen and spikelets (Fig. 1c and Supplementary Fig. 5). In contrast, when the three transgenes were pyramided together by a cross between the homozygous *S1A4*–*S1A6*^t^ and *S1TPR*^t^ lines, the pollen and spikelets of the transgenic F_1_ plants were semi-sterile (Fig. 1d). Furthermore, abnormal embryo sacs were observed in the F_1_ plants of *S1A4*–*S1A6*^t^ × *S1TPR*^t^ (Supplementary Fig. 6), consistent with previous reports in Asian–African rice hybrids^23,31^. These results suggested that these three genes are necessary and sufficient to constitute a gamete-killer system.

We further analyzed the segregation of the *S1A4*–*S1A6*^t^ and *S1TPR*^t^ transgenes. The segregation of *S1TPR*^t^ and *S1A4*–*S1A6*^t^ in their T_1_ generations fit the 1:2:1 ratio, consistent with the hypothesis that the HS requires all three components (Fig. 1e, Supplementary Tables 3 and 4). As expected, the F_1_ of the *S1A4*–*S1A6*^t^ × *S1TPR*^t^ cross (sporophytic genotype *S1A4*–*S1A6*^t^–/*S1TPR*^t^–) was semi-sterile. Moreover, the segregation of *S1TPR*^t^ was severely distorted, with most (75.8%) of the F_2_ individuals carrying homozygous *S1TPR*^t^ and the rest containing hemizygous *S1TPR*^t^ (Fig. 1e and Supplementary Table 5). This indicated that the gametophytic genotype is what matters for the transmission advantage of *S1TPR*^t^. However, the segregation ratio of *S1A4*–*S1A6*^t^ in the F_2_ progeny of *S1A4*–*S1A6*^t^ × *S1TPR*^t^ fit the 1:2:1 ratio, similar to the segregation of *S1TPR*^t^ and *S1A4*–*S1A6*^t^ in their T_1_ generations (Fig. 1e, Supplementary Tables 3–5). Since *S1A4*–*S1A6*^t^ and *S1TPR*^t^ are not linked, this is consistent with *S1TPR*^t^ in the gametophyte providing protection from the sterile effect of *S1A4*–*S1A6*^t^ and *S1TPR*^t^ in the sporophyte. In contrast to the situation in the native *S1-g* allele, here the transgenes are unlinked and the distortion of *S1TPR*^t^ segregation does not affect the normal *S1A4*–*S1A6*^t^ segregation. Moreover, it is consistent with the effect of *S1A4*–*S1A6*^t^ and *S1TPR*^t^ acting in the sporophyte.

These observations prompted us to propose a model using a Punnett square to explain the results of this genetic analysis (Fig. 1f). We reasoned that *S1A4*, *S1TPR*, and *S1A6* might act together in the sporophyte to produce a detrimental sterility signal that kills the male and female gametes. However, the *S1TPR*^t^ transgene alone is capable of protecting the corresponding gametes (with or without *S1A4*–*S1A6*^t^) from the sterility, thereby rescuing the gametes harboring *S1TPR*^t^ and leading to severely distorted segregation favoring *S1TPR*^t^ in the F_2_ progeny but not affecting segregation of the unlinked *S1A4*–*S1A6*^t^.

### *S1TPR* is required for killer and protector function

**Fig. 2:**
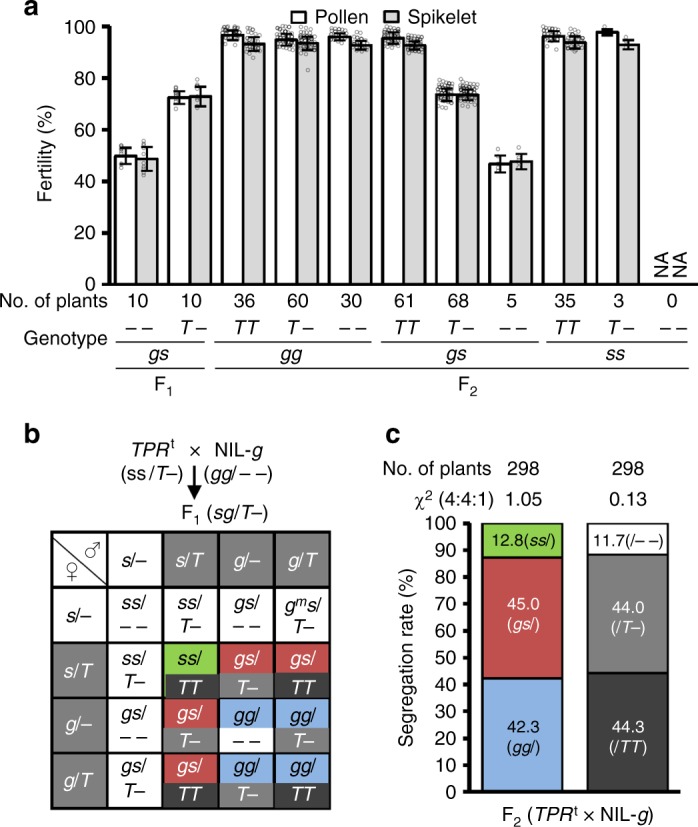
The *S1TPR*^t^ rescues the gametes carrying *S1-s* in hybrids. **a** Pollen and spikelet fertilities of the F_1_ plants derived from the cross between the hemizygous *S1TPR*^t^ (*TPR*^t^, *ss*/*T*–) and NIL-*g* (*gg*/– –), and various genotypes of the F_2_ segregants. Error bars indicate S.D. NA, not available. **b** A proposed model for the segregation behavior of the endogenous *S1* alleles and *TPR*^t^ in the F_2_ plants derived from *TPR*^t^ × NIL-*g*. The F_1_ male and female gametes containing *S1-g* and/or *T* are considered fertile, and those with *S1-s* (*ss*/– –) but lacking *T* are generally sterile. Thus, the expected segregation ratios for *gg*:*gs*:*ss* and *TT*:*T*–:– – are 4:4:1. The color codes are consistent with the genotype of the individuals in the F_2_ population. Black represents homozygous *TPR*^t^; gray represents hemizygous *TPR*^t^; white represents lacking *TPR*^t^; green represents homozygous *S1-s*; red represents heterozygous *S1*; blue represents free homozygous *S1-g*. **c** The segregation rates of the *S1* alleles and the transgene (*T*) in the analyzed F_2_ population fit the expected ratio. Source data of (**a**, **c**) are provided as a Source Data file

If *S1TPR* in the gamete is sufficient to provide the protector function at the *S1* locus, gametes containing the *S1-s* allele should be partially rescued by the *S1TPR* transgene. To test this, we crossed the hemizygous *S1TPR*^t^ line with NIL-*g* to produce F_1_ hybrids (*S1-gS1-s*/*S1TPR*^t^–) that contain the *S1-g* allele in the sporophytic tissue (and thus should activate HS), but will segregate the *S1TPR*^t^ transgene in the gametes. Indeed, the F_1_ and F_2_ plants that are heterozygous for *S1* but lack the *S1TPR*^t^ transgene were semi-sterile (~50%). By contrast, the fertilities of the pollen and spikelets of the F_1_ and F_2_ plants that are heterozygous for *S1* and carry the *S1TPR*^t^ transgene in a hemizygous condition increased to ~75%. Moreover, the *S1*-heterozygotes with homozygous *S1TPR*^t^ were fully fertile (Fig. 2a and Supplementary Table 6), indicating that the *S1TPR*^t^ allows transmission of the *S1-s* allele carrying this transgene. The F_2_ plants homozygous for *S1* (*S1-sS1-s* or *S1-gS1-g*) were fully fertile regardless of whether they contained *S1TPR*^t^ or not (Fig. 2a and Supplementary Table 6). This suggests that *S1TPR*^t^ indeed rescued the gametes containing *S1-s* in a gametophytic manner.

Based on these findings and our hypothesis, we proposed that in the F_1_ plants (*S1-gS1-s*/*S1TPR*^t^–) of *S1TPR*^t^ × NIL-*g*, the *S1-g* allele produced a sterility signal in the sporophytic cells. However, all the *S1-g-*containing gametes survive due to the presence of the endogenous *S1TPR*, and the *S1-s-*containing gametes are aborted unless they carried the *S1TPR*^t^ transgene. The segregation ratios for the genotypes of *S1* (*S1-gS1-g*:*S1-gS1-s*:*S1-sS1-s*) and *S1TPR*^t^ (*S1TPR*^t^*S1TPR*^t^:*S1TPR*^t^–:– –) were therefore predicted to be 4:4:1 in the F_2_ population (Fig. 2b). Indeed, this segregation ratio perfectly fit the ratio from genetic analysis of the *S1TPR*^t^ × NIL-*g* F_2_ population (Fig. 2c and Supplementary Table 6).

**Fig. 3:**
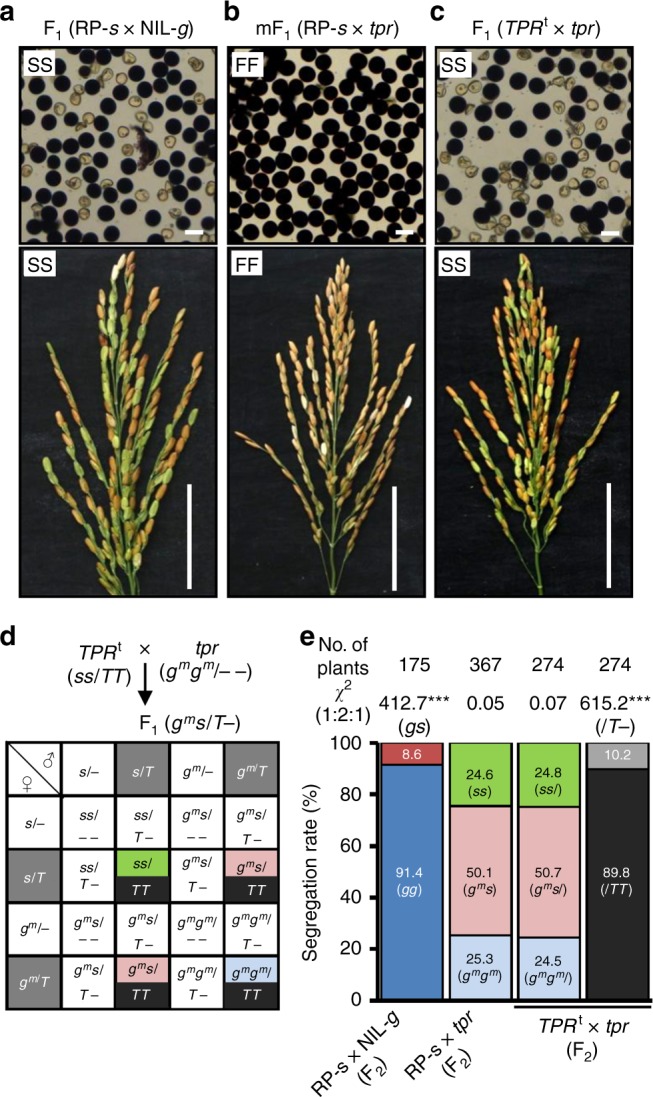
Dual functions of *S1TPR* in *S1* HS. **a** F_1_ hybrids from crossing RP-*s* with NIL-*g* exhibited typical semi-sterile pollen and spikelets. **b** The pollen and spikelets of the mutant F_1_ (mF_1_) plants from crosses between RP-*s* and the CRISPR-knockout mutant *s1tpr* (*tpr*) in NIL-*g* (*g*^*m*^*g*^*m*^) were fully fertile. **c** The F_1_ plants from a cross between the *S1TPR*^t^ (*TPR*^t^, *ss*/*TT*) and *tpr* (*g*^*m*^*g*^*m*^) lines exhibited semi-sterile pollen and spikelets. Bars in (**a**–**c**) represent 50 μm for pollen and 5 cm for panicles. **d** A proposed model for the segregation of the *S1* alleles and transgenes in the F_2_ progeny of the *TPR*^t^ × *tpr* cross. The F_1_ male and female gametes containing the *T* allele (gray background) are fertile, and those without *T* (white background) generally abort. The color codes are consistent with the genotype of the individuals in the F_2_ population. Black represents homozygous *TPR*^t^; green represents homozygous *S1-s*; pink represents heterozygous mutated *S1*; light blue represents homozygous mutated *S1-g*^*m*^. **e** Segregation rates of the *S1* alleles and transgenes (*T*) in the F_2_ plants shown in (**a**–**c**). ***P *<* 0.001 in the *Χ*^2^ test. Source data of (**e**) are provided as a Source Data file

To further verify that *S1TPR* also participates in the killing process, we crossed the homozygous *S1TPR*^t^ plants with the *s1tpr* mutant (the function of *S1TPR* was completely knocked out, resulting in homozygous mutated *S1-g*^*m*^)^27^. We reasoned that if the *S1TPR*^t^ transgene can function together with the endogenous *S1A4* and *S1A6* (still present at the *S1-g*^*m*^ allele) genes to kill the gametes, gamete abortion would be observed in the resultant F_1_ hybrids. As expected, the F_1_ plants exhibited semi-sterile pollen and spikelets, like those of RP-*s* × NIL-*g* (Fig. 3a–c). Since *S1TPR* makes up part of the killer system, and has another role in the protection of *S1TPR*-containing gametes, we therefore hypothesized a segregation model, in which the hybrids (*S1-g*^*m*^*S1-s*/*S1TPR*^t^–) produce a detrimental sterility signal (in a sporophytic manner) to kill the gametes carrying *S1-s* or *S1-g*^*m*^, but the *S1TPR*^t^-containing gametes survive (Fig. 3d). As expected, in the F_2_ population of the *S1TPR*^t^ × *s1tpr* cross the segregation ratio of the *S1* genotypes (*S1-g*^*m*^*S1-g*^*m*^:*S1-g*^*m*^*S1-s*:*S1-sS1-s*) fit the 1:2:1 ratio, and the *S1TPR*^t^ genotypes had a significantly distorted segregation ratio (Fig. 3e and Supplementary Table 7). These results also suggested that the abortion of the *S1-s* or *S1-g*^*m*^-containing gametes in the hybrids is independent of the *S1TP* gene at *S1-s* or other gametophytic responder genes.

### S1A6-S1A4-S1TPR is a tripartite complex in the nucleus

**Fig. 4:**
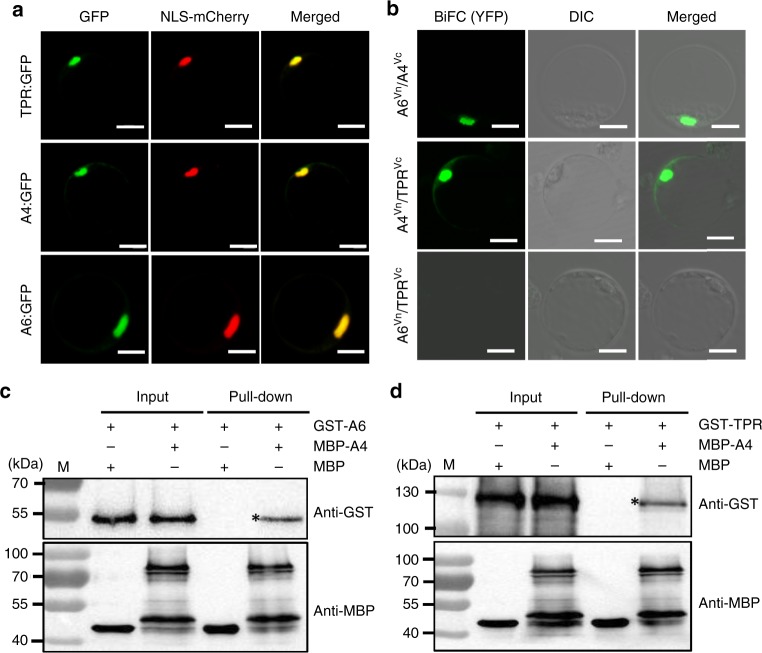
S1TPR-S1A4–S1A6 is a tripartite complex in the nucleus. **a** S1TPR (TPR, top), S1A4 (A4, middle), and S1A6 (A6, bottom) were fused with GFP and each was co-expressed with the nuclear localization signal marker (NLS-mCherry) in the rice protoplasts. The GFP fluorescence perfectly matched the mCherry fluorescence. Scale bars, 10 μm. **b** BiFC assays showed that, in the nuclei of rice protoplasts, A4 interacts with A6 and TPR (top and middle), but A6 does not interact with TPR (bottom). Scale bar, 10 μm. **c**, **d** Pull-down assays confirmed that A4 interacts with A6 (**c**) and TPR (**d**) in vitro. “*” indicates the MBP-A4 pull-down proteins (GST-A6 or GST-TPR) detected using the anti-GST antibody. M means 10–180 kDa protein size marker (Thermo Fisher Scientific, CA, USA). Source data of (**c**, **d**) are provided as a Source Data file

In this gamete killer–protector system, *S1A6* encodes a smaller peptidase with similar features to the S1TPR peptidase^23,27^, and *S1A4* encodes an uncharacterized protein of 261 amino acids without any putative conserved domains (Supplementary Fig. 7). To study the subcellular localization of these proteins, *S1A4*, *S1A6*, and *S1TPR* were fused with the *GFP* gene sequence, respectively, and expressed in rice protoplasts. All these proteins localized in the nucleus (Fig. 4a), suggesting that they may interact with each other in the nucleus. Consistent with this, bimolecular fluorescence complementation (BiFC) and pull-down assays indicated that S1A4 interacted with S1A6 and S1TPR respectively in the nucleus, but S1TPR did not interact with S1A6 directly (Fig. 4b–d).

### Possible biochemical effect of the *S1*-HS system

Given that the *S1* HS factors interact in the nucleus, we next examined their effects on the transcriptome. To determine which biochemical processes cause gamete abortion and to explore the possible mechanisms by which this occurs, we sequenced the transcriptomes of anthers (at the microspore mother cell stage to meiosis stage) from RP-*s*, NIL-*g*, and their F_1_ hybrids. We identified 250 commonly upregulated genes and 74 commonly downregulated genes in the NIL-*g* and F_1_ plants relative to their levels in RP-*s* (Supplementary Fig. 8a). These genes were then analyzed using the KEGG pathway database (http://www.genome.jp/kegg/), revealing that the expressions levels of genes involved in photosynthesis and the degradation of valine, leucine, and isoleucine (branched-chain amino acids, BCAAs) were significantly upregulated in the NIL-*g* and F_1_ plants versus the RP-*s* plants (Supplementary Figs. 8b and 9). However, the analysis did not identify any significantly enriched pathway among the downregulated genes. The expression profiles of these differentially expressed genes involved in photosynthesis and BCAA degradation were further confirmed using qRT-PCR (Supplementary Fig. 10). These results hinted that photosynthesis and BCAA may be associated with gamete development.

### Evolutionary origin of the *S1* gamete killer–protector system

According to the BDM model, hybrid incompatibility is caused by a detrimental interaction between the divergent alleles of two independent lineages, which may have been derived from a recent common ancestor^4^. To trace the evolution of the *S1* locus, we first used the *S1TPR*, *S1TP*, *S1A4*, and *S1A6* nucleotide coding sequences to perform a BLAST search for putative orthologs in the *Poaceae* sequences using the GenBank database (https://www.ncbi.nlm.nih.gov/), and constructed a phylogenetic tree of the candidates. We found that the nucleotide coding sequences of *S1TPR*, but not *S1A4* and *S1A6*, were significantly similar to rice in the genomes of *Zea mays*, *Sorghum bicolor*, *Brachypodium distachyon*, *Hordeum vulgare*, *Aegilops tauschii*, *Triticum aestivum*, and *Setaria italica* (Supplementary Fig. 11). Notably, the orthologous gene (LOC101754700) in *S*. *italica* has the highest nucleotide identity (ca. 82%) with *S1TPR* and *S1TP*. Thus, the *S1TPR* ortholog in *S*. *italic* is used as one of the appropriate outgroup references for analyzing the divergence of *S1TPR* and *S1TP*.

To further confirm the point of divergence in the *S1* locus of *Oryza*, we identified the *S1TPR*, *S1TP*, *S1A4*, and *S1A6* sequences in the AA genome species of *Oryza* (*O*. *meridionalis*, *O*. *longsistaminata*, *O*. *barthii*, *O*. *rufipogon*, *O*. *glaberrima*, and *O*. *sativa*) and several non-AA genome species, including *O*. *officinalis* (CC genome), *O*. *rhizomatis* (CC genome), *O*. *eichingeri* (CC genome), and *O*. *minuta* (BBCC genome), which are all closely related to the AA genome *Oryza* species based on molecular evidence^33^. These non-AA genome species were also used as outgroups in the divergent analysis of the genes. We found that only *S1TPR* and/or *S1TP*, but not *S1A4* and *S1A6*, were present in these outgroup species, indicating that *S1A4* and *S1A6* likely newly evolved in the *Oryza* species with AA genomes (Supplementary Fig. 12 and Supplementary Data 1).

Since seven single-nucleotide polymorphisms (SNPs, sites 1–7) are present between the *S1TPR* and *S1TP* coding sequences (Supplementary Fig. 12)^27^, we further analyzed the patterns of these seven SNPs in 443 accessions of the AA-genome *Oryza* species and some other outgroup species (including *S*. *italica*) to trace the sequence divergence of the *S1TPR* and *S1TP* genes in these species. An *S1TPR*-type allele (Allele 1) was detected in *S*. *italica*, *O*. *officinalis*, *O*. *minuta*, *O*. *longsistaminata*, and *O*. *meridionalis* (Supplementary Fig. 12 and Supplementary Data 1), thus likely representing the primitive form of *S1TPR*. Notably, the nucleotides at SNP site 7 (C to A variation causing the premature stop codon in *S1TP*) were polymorphic not only among the AA genome species, but also among the analyzed CC genome species. In contrast, the polymorphisms of the SNP sites 1–6 were present only in AA genome species (Supplementary Fig. 12 and Supplementary Data 1), suggesting that SNP 7 in *S1TPR* and *S1TP* arose early in the evolution of the *Oryza* genus and that various alleles co-existed in the primitive *Oryza* gene pool.

On the basis of these detected SNP patterns, we identified at least fifteen *S1TP* alleles (Alleles 1–1 to 1–15) that contained variations at the SNP sites 1, 3, 4, 6, and 7 in AA-genome *Oryza* species, including *O*. *longistaminata*, *O*. *meridionalis*, *O*. *rufipogon*, and *O*. *sativa*. All *O*. *rufipogon* accessions (68) and all *O*. *sativa* accessions (116) carried the one-gene *S1* structure containing *S1TP* (haplotype). Furthermore, we found four *S1TPR* alleles (Alleles 2-1 to 2–4) that carried variations at the SNP sites 2 and 5 in *O*. *barthii* and *O*. *glaberrima* (Supplementary Fig. 12).

**Fig. 5:**
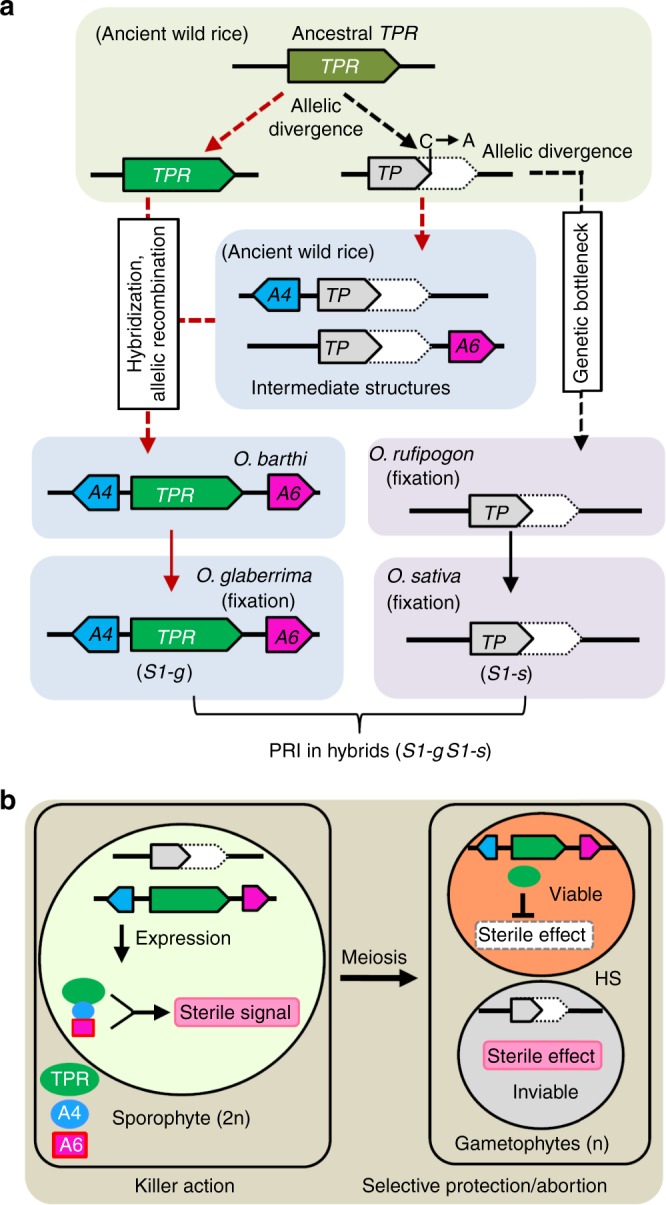
Evolution and mechanism of the *S1* killer–protector system. **a** A simplified evolutionary trajectory of the *S1* alleles in *Oryza*. Current *S1TPR* (*TPR*) and *S1TP* (*TP*) alleles in the *Oryza* genus might be derived from the ancestral *TPR* genes from two independent lineages. In one lineage, the diverged *TP* alleles passed through a bottleneck and migrated into *O*. *rufipogon*, eventually being fixed as *S1*-*s* in *O*. *sativa*. In another lineage, the intermediate structures (*A4*-*TP* and *TP*-*A6*) carrying the new genes *S1A4* (*A4*) and *S1A6* (*A6*) arose in ancient wild rice, and the *A4*-*TPR-A6* three-gene structure were generated in *O*. *barthii*, probably by natural hybridization and allelic recombination; this structure further migrated into *O*. *glaberrima* as the functional *S1-g* allele. In hybrids between *O*. *glaberrima* and *O*. *sativa*, this gamete killer–protector system causes postzygotic reproductive isolation (PRI). **b** A working model for the *S1* gamete killer–protector system in African-Asian rice hybrids. In the sporophytic cells (megaspore/microspore mother cells) of the hybrids, the three-protein complex, comprising TPR, A4, and A6, expressed from *S1-g* may produce a sterility-trigging signal. This signal is retained in the post-meiotic male and female gametes and causes the selective abortion of gametes carrying *S1-s*, whereas TPR in the *S1-g* gametes eliminates the sterility. *S1-g* therefore has a strong transmission advantage in the hybrids and acts as a typical ultra-selfish genetic element

The non-overlapping natural variations in the *S1TPR* and *S1TP* alleles between *O*. *glaberrima* and *O*. *sativa* suggested that at least two independent lineages evolved from the common ancestral lineage carrying the ancestral *S1TPR* gene (Fig. 5a, Supplementary Figs. 12 and 13). In one lineage, the resultant *S1TP* variants (Alleles 1-1 to 1–16) passed a bottleneck and some (Alleles 1–3 to 1–8) were transmitted into *O*. *rufipogon*, resulting in the eventual fixing of two (Alleles 1–3 and 1–8) as the current *S1*-*s* in *O*. *sativa*; this allele is present in all populations of *O*. *sativa* (Fig. 5a, Supplementary Figs. 12 and 13). By contrast, *S1-g* likely evolved in another lineage (Fig. 5a, Supplementary Figs. 12 and 13). Given that *S1A4* and *S1A6* are absent in the CC and BBCC genomes of other *Oryza* species, we speculated that the *S1A4*-*S1TPR*/*S1TP-S1A6* alleles might newly evolve in the AA-genome *Oryza* species. Consistent with this hypothesis, three types of *S1TP*-containing structures with the genes *S1A4* and/or *S1A6*, inserted upstream and downstream of *S1TP*, respectively, were identified in *O*. *meridionalis* (Fig. 5a, Supplementary Figs. 12 and 13). Seven accessions had the *S1A4-S1TP* structure (Alleles 1–9 to 1–11), five accessions had the *S1TP-S1A6* structure (Alleles 1–12 and 1–13), and two accessions had the *S1A4-S1TP-S1A6* structure (Alleles 1–14 and 1–15) (Fig. 5a, Supplementary Figs. 12 and 13). Similarly, three types of *S1TPR*-containing structures with *S1A4* and/or *S1A6* (Alleles 2–2 to 2–4) were identified in *O*. *barthii*, the wild progenitor of *O*. *glaberrima* (Fig. 5a, Supplementary Figs. 12 and 13): 10 accessions had the *S1A4-S1TPR* structure, one accession had the *S1TPR-S1A6* structure, and 52 accessions had the three-gene structure *S1A4-S1TPR-S1A6* (Fig. 5a, Supplementary Figs. 12 and 13).

Despite the incomplete lineage sorting in the AA-genome *Oryza* species, *O*. *meridionalis* is considered to be the sister group of the other AA species^33,34^. Intermediate variants containing *S1TP* and the flanking genes *S1A4* and/or *S1A6* (*S1A4*-*S1TP*, *S1TP*-*S1A6*, *S1A4*-*S1TP*-*S1A6*) may therefore have appeared first in *O*. *meridionalis*, followed by the two-gene intermediate structures carrying *S1TPR* (*S1A4-S1TPR* and *S1TPR-S1A6*) and the final functional *S1A4*-*S1TPR*-*S1A6* complex (Fig. 5a and Supplementary Fig. 13). These complexes probably arose during allelic recombination events in natural hybrids between ancestral species possessing the intermediate structures (*S1TPR*/*S1A4-S1TP*, *S1TPR*/*S1TP-S1A6*, and *S1A4-S1TPR*/*S1TPR-S1A6*) (Fig. 5a and Supplementary Fig. 13), finally generating *S1A4-S1TPR-S1A6* in the *O*. *barthii* lineage.

All 133 analyzed accessions of *O*. *glaberrima* were found to possess the complete *S1A4-S1TPR-S1A6* structure, the same allele (Allele 2–4) was detected in *O*. *barthii*, indicating that the *S1-g* allele had been fixed in African rice (Fig. 5a, Supplementary Figs. 12 and 13). In addition, 13 accessions of *O*. *meridionalis* possessed another structure of *S1A4*-*S1TPR*-*S1A6*, where *S1TPR* is the primitive form of Allele 1 (Supplementary Figs. 12 and 13).

### A working model for the *S1*-mediated killer–protector system

Our results revealed that the three closely linked genes *S1A4*, *S1TPR*, and *S1A6* at the *S1-g* allele constitute a tripartite gamete killer–protector complex that acts as an ultra-selfish genetic complex generating a sterility signal via physical interaction of the three encoded proteins in the sporophytic cells. S1TPR also serves as a protector in *S1-g* gametophytic cells (Fig. 5b). In *O*. *glaberrima* plants with a homozygous *S1-g* allele, all gametes escape abortion because S1TPR eliminates the detrimental effect induced by *S1-g*. The *S1-s* allele in *O*. *sativa* harbors only the defective *S1TP* gene and lacks the functional gamete killer and protector; therefore, all gametes are viable. In the interspecific hybrids, the tripartite *S1A4*-*S1TPR*-*S1A6* complex of *S1*-*g* causes the selective abortion of gametes containing the *S1-s* allele, resulting in a transmission advantage for *S1*-*g* (Fig. 5b).

## Discussion

To date, there are two genetic models for HS in rice^19^. The HS loci *S5*, *S7*, *Sa*, *Sc*, and *qHMS7* fit the one-locus model^8,12,14,15,29^, while *S27*/*S28*, *DPL1*/*DPL2*, and *DGS1*/*DGS2* fit the two-locus model^25,26,28^. There is emerging molecular evidence to support the conclusion that the loci involved in one-locus HS systems are usually complex loci, comprising multiple adjacent and functionally related genes, as we previously reported for the *Sa* locus containing the adjacent genes *SaF* and *SaM*^8^. In the *Sa* and *S5* systems, the symmetric allelic HS interactions are represented by the molecular interactions of the proteins from both alleles of the parental lines^8,14^; for example, the interacting proteins in the three-component *Sa* complex, SaF^+^ and SaM^+^, are contributed by the *indica* allele, while the SaM^–^ protein is encoded by the *japonica* allele^8^. Similarly, in the *S5* gamete-killer system, the ORF5+ protein is encoded by the *indica* allele, but ORF4+ is contributed by the *japonica* allele^14^.

*S1* is considered the predominant HS locus found in *Oryza*, because of its strong genetic effect on HS of male and female gametes in interspecific hybrid progenies. In this study, we showed that the functional African rice allele, *S1-g*, consists of three closely linked actively expressed genes *S1A4*, *S1TPR*, and *S1A6* (*SSP*) (Fig. 5b). The gamete-killer function of the *S1* HS system requires all the three components (S1A4, S1TPR and S1A6) only from *S1-g*, while the protector function depends on solely on *S1-g-*derived S1TPR; no component from the Asian rice allele is required for this gamete killer–protector system. The *S1* gamete killer–protector system is therefore determined by the African rice allele, representing an asymmetric allelic interaction. This characteristic is distinct from the *S5* and *Sa* systems, which are symmetric allelic interactions involving components from both divergent alleles. The *S1TPR* has dual roles in killer and protector function, which is distinguished from the reported killer–protector systems^14,15^, in which killer and protector are conferred by different factors. Thus, this *S1* gamete killer–protector system expands our understanding of the single-locus BDM model.

According to our transcriptome sequencing analysis, six genes in the BCAA degradation pathway were expressed at significantly lower levels in the RP-*s* plants than in the F_1_ and NIL-*g* plants (Supplementary Figs. 8–10). Dysfunctional BCAA biosynthesis is known to cause the abortion of both male and female gametophyte development^35^. Therefore, we propose that the complex of the three *S1-g* gene products may induce excessive BCAA degradation in the F_1_ and NIL-*g* plants, resulting in the sterility effect that affects gamete development. The S1TPR produced in *S1-g*-type gametes may result in adequate levels of BCAA via its peptidase function, as it resembles trypsin peptidase in animals^36^. The gametes carrying *S1-s* in the F_1_ hybrids lacking functional S1TPR would fail to restore fertility due to their BCAA deficiency. In Asian rice cultivars (*S1-sS1-s*), the genes involved in BCAA degradation are expressed at low levels, meaning that the gametes have enough BCAA to continue their development. In addition, we reasoned that the upregulation of photosynthesis-related genes in the F_1_ and NIL-*g* plants may be the result of a regulatory feedback loop monitoring nutrient or energy deficiency (Supplementary Figs. 8 and 10), which is an interesting topic for future study.

The hypothesized Gondwanaland origin of the *Oryza* genus explains the extensive geographic distribution of the *Oryza* in modern species^17,18,37^. However, the current divergence times estimated from the molecular evolution between species in the *Oryza* genus are not in accordance with this theory^34,38^. These divergence times were based on sequencing analysis of the currently available species, which may cause artifacts or bias in the algorithm used due to the inability to sequence extinct ancestral species. If the *Oryza* genus did indeed originate in Gondwanaland, we can hypothesize that the *S1* alleles in Asian rice and African rice might have originated from a common progenitor in Gondwanaland, and later evolved in parallel but independent lineages after their geographical separation (Fig. 5a). The ancient *Oryza* populations located on the ancient Australian continent might have been separated from the majority of the *Oryza* taxa due to the breakup of Gondwanaland and subsequent continental drift, causing the *S1TP* allele variants (without *S1A4* or *S1A6*) to pass through a genetic bottleneck before entering the *O*. *rufipogon* and *O*. *sativa* lineages in Asia. Nevertheless, other intermediate structures of *S1TPR* and/or *S1TP* variants carrying *S1A4* and/or *S1A6* in the ancestral *Oryza* species might have continued to evolve on the ancient supercontinent. The functional *S1A4*-*S1TPR*-*S1A6* structure was eventually generated in the *O*. *barthii* lineage and was further transferred into *O*. *glaberrima* and fixed on the African continent. The origin and evolution of the *S1* alleles, from the intermediate (neutral) haplotypes (*S1A4-S1TPR* and *S1TPR-S1A6*) to the functional ultra-selfish genetic complex, might therefore be associated with or contribute to the speciation of the related *Oryza* species.

Polymorphism has been shown to be inevitable in the progression from allele origination to fixation at BDM loci^4^, consistent with the formation of the *S1* HS allele (Supplementary Figs. 12 and 13 and Supplementary Data 1). Extensive sequence variations were observed in *S1TPR* and *S1TP* at the *S1* locus in wild rice species (Supplementary Figs. 12 and 13 and Supplementary Data 1), allowing us to propose that their common ancestral sequences may be polymorphic in the *Oryza* species. Although the outline of the *Oryza* phylogeny is clear, the exact relationships among the *Oryza* species are elusive due to discordance between the phylogenetic trees for different genes. These issues are caused by factors such as incomplete lineage sorting^34,39,40^, meaning that the *S1* alleles in Asian rice and African rice might have originated from an unknown common progenitor. The geographical distribution of polymorphisms at the *S1* locus might have arisen from the long-distance dispersal of these species, which was followed by selection and fixation.

Besides *S1* described in this study, several other complex HS loci that are composed of two or three closely linked HS genes (such as *Sa*, *S5* and *qHMS7*) have been identified in *Oryza* species^8,14,15^. These findings suggest that during speciation, generation of such complex HS loci may have advantages such as simple inheritance (as single functional genetic units having minimum recombination between the closely linked genes/alleles) and maximum genetic effect for postzygotic reproductive isolation.

The functional *S1-g* system is predominantly fixed in *O*. *glaberrima* populations, which explains why researchers failed to identify natural hybrid-compatible (neutral) *S1* alleles that could be used to break down the reproductive barrier in interspecific crosses between *O*. *glaberrima* and *O*. *sativa*. Our findings suggest that artificial hybrid-compatible *S1* alleles could be created for the utilization of distant heterosis by disrupting any one of the three genes in *S1-g* by CRISPR/Cas9 knockout^12,27,41^. As an alternative strategy, replacing the premature stop codon in *O*. *sativa* cultivars, using base editing^42^ would rescue *S1TP* to functional *S1TPR* and thereby allow gamete normal development in interspecific hybrids.

## Methods

### Plant materials

A near-isogenic line NIL-*g* containing *S1*-*g* was developed using an African rice (*O*. *glaberrima*) line IRGC102203 as the *S1* donor and an Asian rice (*O*. *sativa*) line IRAT216 containing *S1-s* as the recurrent parent (RP-*s*).

The genomic sequences of *S1TPR*, *S1A4*, and *S1A6* were amplified by specific primers from BAC OG-BBa0049I08, which was kindly provided by Dr. Rod A. Wing (the University of Arizona). The genomic sequences were sub-cloned into the binary vector pCAMBIA1300 using a Gibson assembly assay to generate the functional complementation constructs^43^. The target sites were fused with sgRNA expression cassettes and sub-cloned into the CRISPR/Cas9 constructs^44^ for knocking out genes *S1A2*–*S1A6*. All functional complementation constructs and knockout plasmids were transformed into RP-*s* and NIL-*g*, using the *Agrobacterium tumefaciens-*mediated method. The primers used for vector construction are listed in Supplementary Table 8.

### Phenotyping of pollen and spikelet fertility

The plant materials were grown in Guangzhou, China during the normal growing season and in Sanya, China during the winter season. The anthers of 3–5 mature flowers from each independent flowering individual were stained with I_2_-KI solution to enable the observation and imaging of pollen fertility using a light microscope (Axio Observer D1, Carl Zeiss, Oberkochen, Germany). When the seeds ripened, the spikelet fertility was examined as the seed-setting rate in the main panicle of each individual.

### Histological analysis

The histological analysis was performed using paraffin sectioning method^23^. Briefly, spikelets of RP-*s*, NIL-*g*, the F_1_ plants from the RP-*s* × NIL-*g* and the F_1_ plants from the *S1A4*–*S1A6*^t^ × *S1TPR*^t^ cross were fixed in FAA (1:1:18, formalin:glacial acetic acid:50% ethanol) for at least 24 h. Their ovules were then dehydrated, embedded in paraplast and cut into 8-μm longitudinal sections. The sections were stained with toluidine blue before being observed using a light microscope.

### Genotyping of transgenes and *S1* alleles in F_2_ populations

The T-DNA flanking sequences of *S1TPR*^t^ and *S1A4*–*S1A6*^t^ were determined using hiTAIL-PCR^45^. Plants were genotyped for their T-DNA tag using PCR with the specific primer sets (Supplementary Table 8). In addition, the genotypes of *S1*-alleles were determined by the *S1*-linked In/Del marker 2170^27^ (Supplementary Table 8).

### Reverse transcription, expression and transcriptome analyses

The anthers, panicles and microspores were collected from PR-*s* and NIL-*g* at different stages and their total RNA were extracted. For each sample, 2 μg of total RNA was reverse transcribed to synthesize the first-strand cDNA according to the manufacturer’s instructions (Toyobo, Osaka, Japan). The quantitative RT-PCR was conducted using gene-specific primers (Supplementary Table 8) with three biological replicates^46^. The data were normalized using *OsActin1* as the endogenous control.

The transcriptome sequencing of the anther samples was performed by GENE DENOVO Co. Ltd (Guangzhou, China). Genes with a significantly different expression level (upregulated by at least 2.82-fold or downregulated by at least 0.35 fold) in the F_1_ and NIL-*g* plants relative to RP-*s* were selected for further analysis. The genes that were commonly upregulated or downregulated in the F_1_ and NIL-*g* plants were identified and analyzed using the KEGG pathway database (http://www.genome.jp/kegg/). The expression patterns of the differentially expressed genes involved in high-scoring pathways were further validated using quantitative RT-PCR.

### Subcellular localization and BiFC assays

The coding sequences of *S1TPR*, *S1A4*, and *S1A6* were cloned into the pLYd1GFP vector carrying the GFP tag to assess their subcellular localization. They were also cloned into pVN and pVC and were fused with the N-terminal or C-erminal sequence of *YFP*, respectively, for the BiFC assay^47^. The plasmids were transfected into rice protoplasts followed by 15 h incubation in dark at 30 °C, after which their fluorescence was imaged using confocal microscopy (LSM 780 DUO, Carl Zeiss, Oberkochen, Germany).

### In vitro protein pull-down assay

The pull-down assays were conducted according to the manufacturer’s instructions (New England Biolabs, MA, USA). Briefly, the S1A4 protein was fused to an MBP-tag as the bait, while the S1A6 or S1TPR proteins were fused with a GST-tag as the prey. The MBP-S1A4, GST-S1A6, or GST-S1TPR proteins were expressed in *Escherichia coli* Rosetta (DE3). The cells containing these recombinant proteins were harvested in phosphate-buffered saline (pH 7.4) and then were ruptured by sonication. The lysates containing MBP-S1A4 were pulled down using amylose resin and mixed with lysates containing GST-S1A6 or GST-S1TPR. The proteins were pulled down and detected using western blotting with anti-MBP (TransGen Biotech, Beijing, China, #HT701, 1:5000 dilution) and anti-GST antibodies (TransGen Biotech, Beijing, China, #HT601, 1:5000 dilution), respectively. Uncropped blots are presented in the Source Data file.

### Phylogenetic and evolutionary analysis of the *S1* locus

To determine the orthologs of *S1A4*, *S1A6*, and *S1TPR*, their nucleotide sequences were used as templates in a BLAST search of the *Poaceae* family in the GenBank database (https://www.ncbi.nlm.nih.gov/). The sequences of the putative orthologs were downloaded and used for the phylogenetic study. The phylogenetic tree was constructed using the maximum likelihood method via MEGA7 (www.megasoftware.net/), with 1,000 bootstrap replications. To trace the ancestral *S1TPR* and *S1TP* sequences, seven non-synonymous SNPs were analyzed in two accessions of *S*. *italica* and 458 rice accessions. For the bioinformatics analysis, the publicly available sequences from the OMAP project^48,49^, the wild rice genome project^50^, the 3000 rice genomes project^51^ and the African wild rice genome^52^ were downloaded from NCBI. The short reads were aligned to the genomic sequences of the *S1-g* and *S1-s* alleles using Burrows-Wheeler Aligner^53^. The depth of coverage and the SNPs were detected using the SAMtools package^54^. To determine the presence of *S1A4* and *S1A6*, coverage was calculated based on their depth at each nucleotide position with a Python program. Primer sets covering the genomic region of *S1TPR*, *S1TP*, *S1A4*, and *S1A6* were designed to amplify the target region for the validation of the sequences in several collected accessions of wild and cultivated rice species to trace the divergence of the *S1* locus.

## Supplementary information

Supplementary Information

Peer Review

Reporting summary

Description of Additional Supplementary Files

Supplementary Data 1

Source data

## Data Availability

Data supporting the findings of this work are available within the paper and its Supplementary Information files. A reporting summary for this Article is available as a Supplementary Information file. The source data underlying Figs. 1e, 2a, 2c, 3e, and 4c, 4d and Supplementary Figs 1b, 2, 4d, 4e, 4f, 4g, 8a, 8b, 10 and Supplementary Tables 2–7 are provided as a Source Data file. The genomic DNA and messenger RNA sequences of gene *S1A4* have been deposited in NCBI GenBank database under accession number MK105813 and MK105814, respectively. The raw data of transcriptome sequencing have been deposited in NCBI Sequence Read Archive under accession number PRJNA540398. All data are available from the corresponding author upon request.
