# Supplementary material for: An asymmetric allelic interaction drives allele transmission bias in interspecific rice hybrids
Source: Nat Commun. 2019 Jun 7;10:2501. doi: 10.1038/s41467-019-10488-3 (PMC6555797; doi:10.1038/s41467-019-10488-3)
Supplement: Supplementary file 1 — Supplementary Information [file 41467_2019_10488_MOESM1_ESM.pdf]

# An asymmetric allelic interaction drives allele transmission bias in interspecific rice hybrids

Xie *et al*

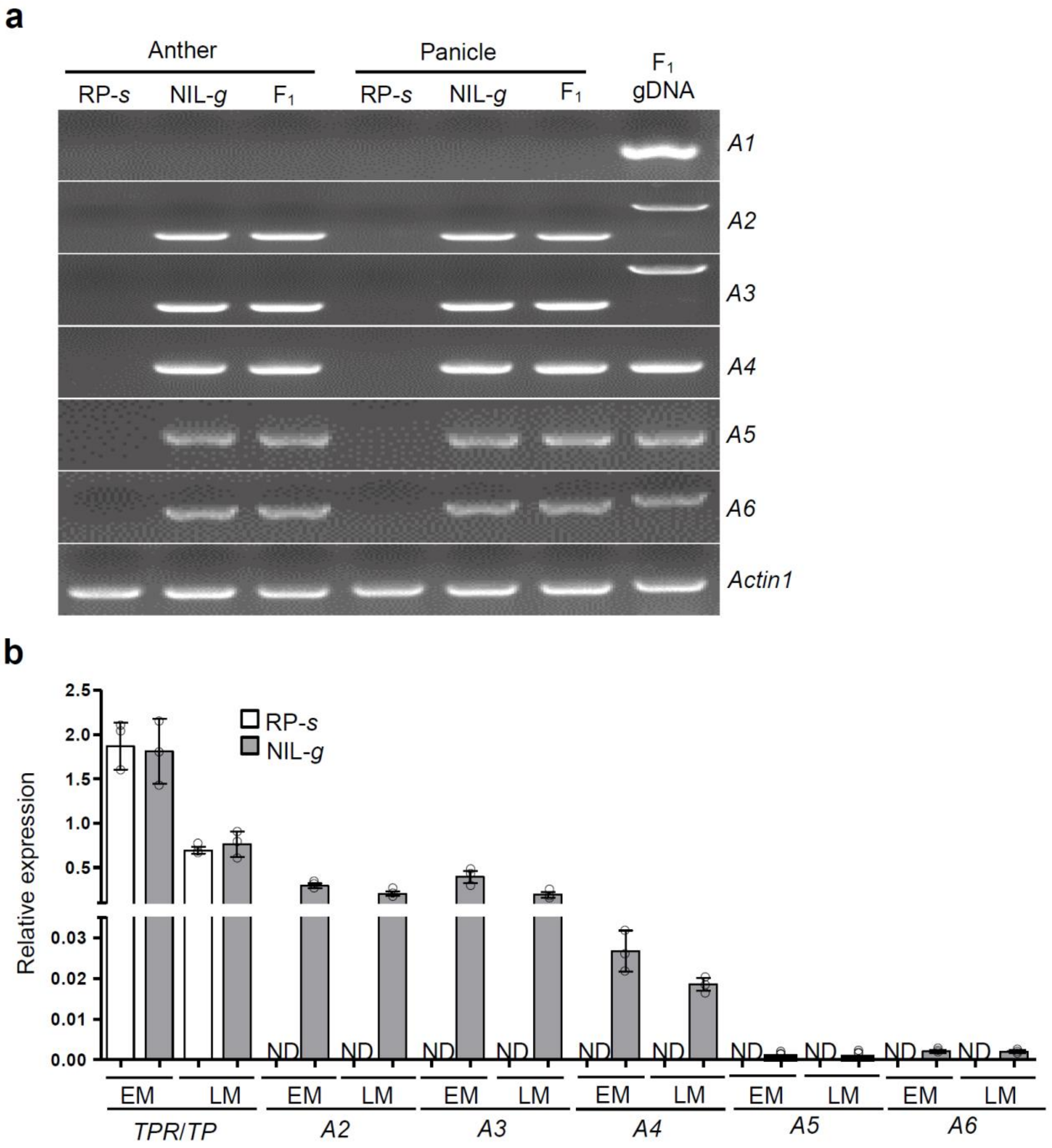


**Supplementary Figure 1**. **Expression analysis of candidate genes at the *S1* locus. (a)** Expression analysis of the candidate genes *S1A1~S1A6* (*A1~A6*) in the anthers and panicles using RT-PCR (32 cycles for target genes and 27 cycles for *OsActin1*). The anthers were at the microspore mother cell stage to meiosis stage. **(b)** Expression analysis of the candidate genes *S1TPR* (*TPR*), *A2~A6* in microspores at different developmental stages using qRT-PCR. ND, not detectable; EM, early microspore stage; LM, late microspore stage. *OsActin1* serves as a reference. Data are mean ± SD ($n$ = 3). Source data of Supplementary Figure 1b are provided as a Source Data file.

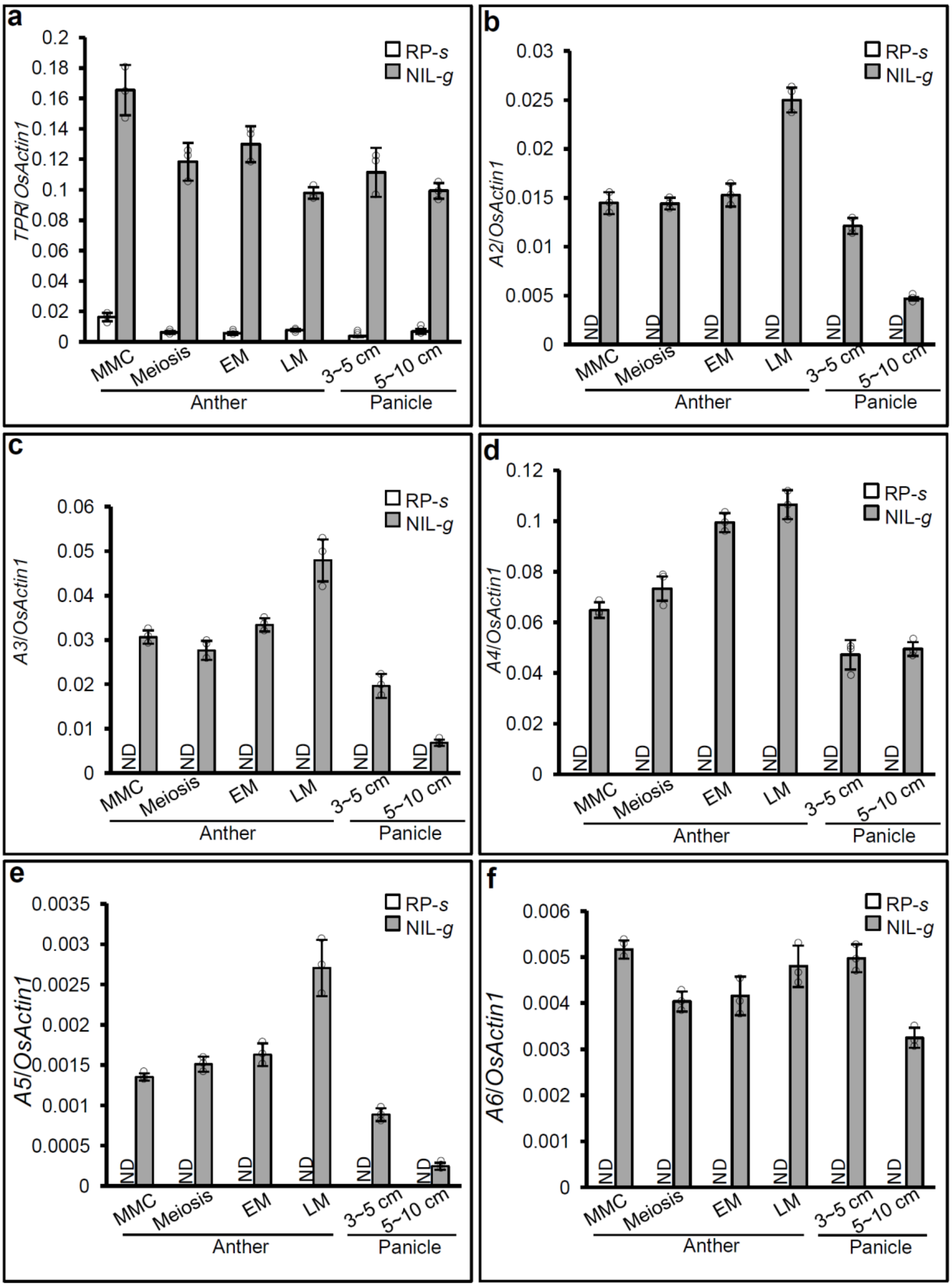


**Supplementary Figure 2**. **Expression analysis of the candidate genes in the *S1-g* mapping region.** Quantitative RT-PCR was used to quantify the expression levels of *S1TPR* (*TPR*) and *S1A2~S1A6* (*A2~A6*) in the anthers and panicles at different reproductive stages, including the microspore mother cell (MMC), meiosis, early microspore (EM), and late microspore (LM), and the young panicles 3~5 cm and

5~10 cm in length. ND, not detectable. Data are mean ± SD ($n$ = 3). Source data are provided as a Source Data file.

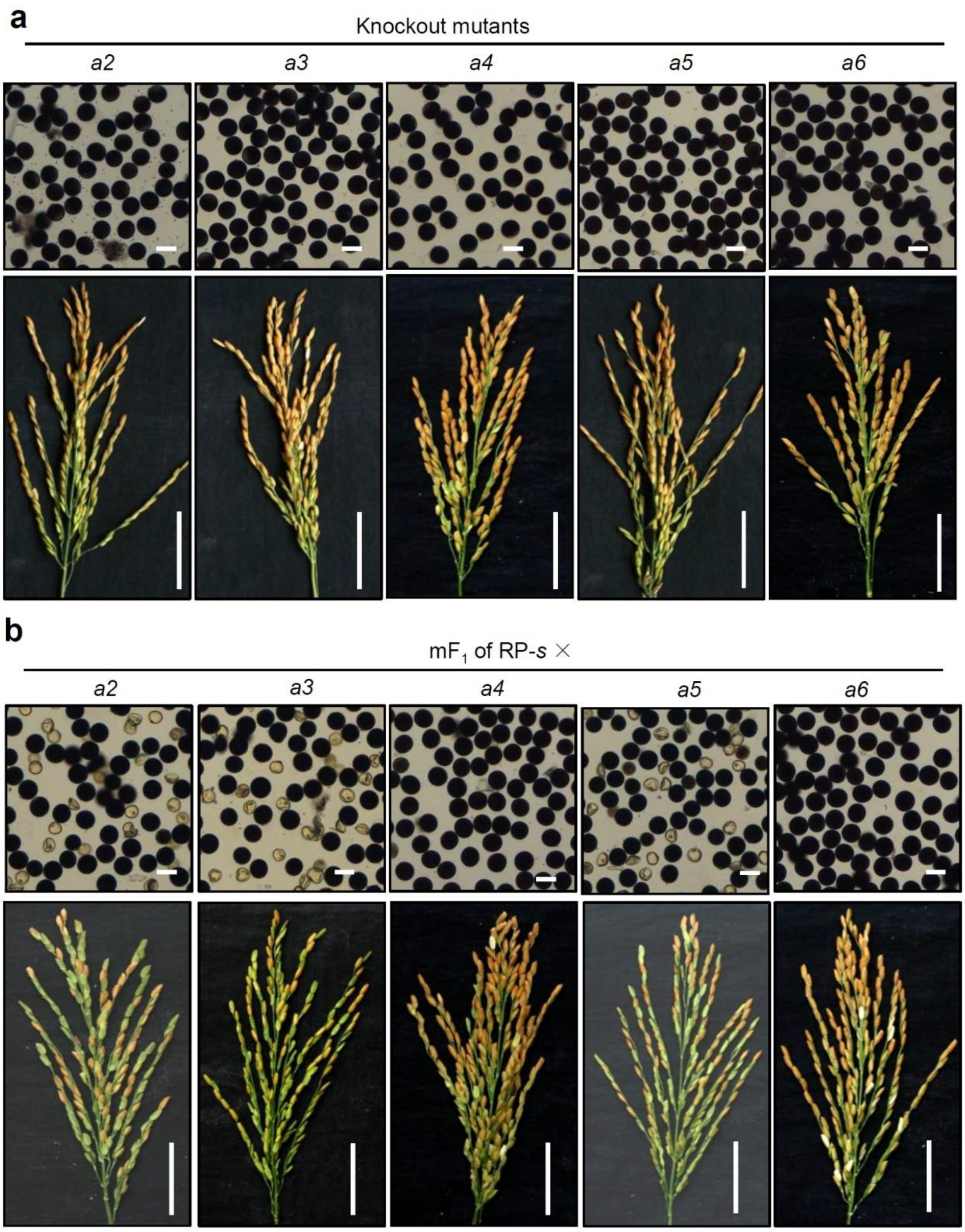


**Supplementary Figure 3**. ***S1A4* and *S1A6* are not essential for gamete development, but required for hybrid sterility. (a)** The pollen and spikelets of CRISPR/Cas9 knockout mutants *s1a2~s1a6* (*a2~a6*) in NIL-*g* had normal fertility. **(b)** The knockout of *S1A4* (*A4*) and *S1A6* (*A6*), but not *S1A2* (*A2*), *S1A3* (*A3*), and *S1A5*

(*A5*), eliminated *S1*-mediated HS in the mutant hybrids (m$F_1$). Bars represent 50 µm for pollen and 5 cm for panicles.

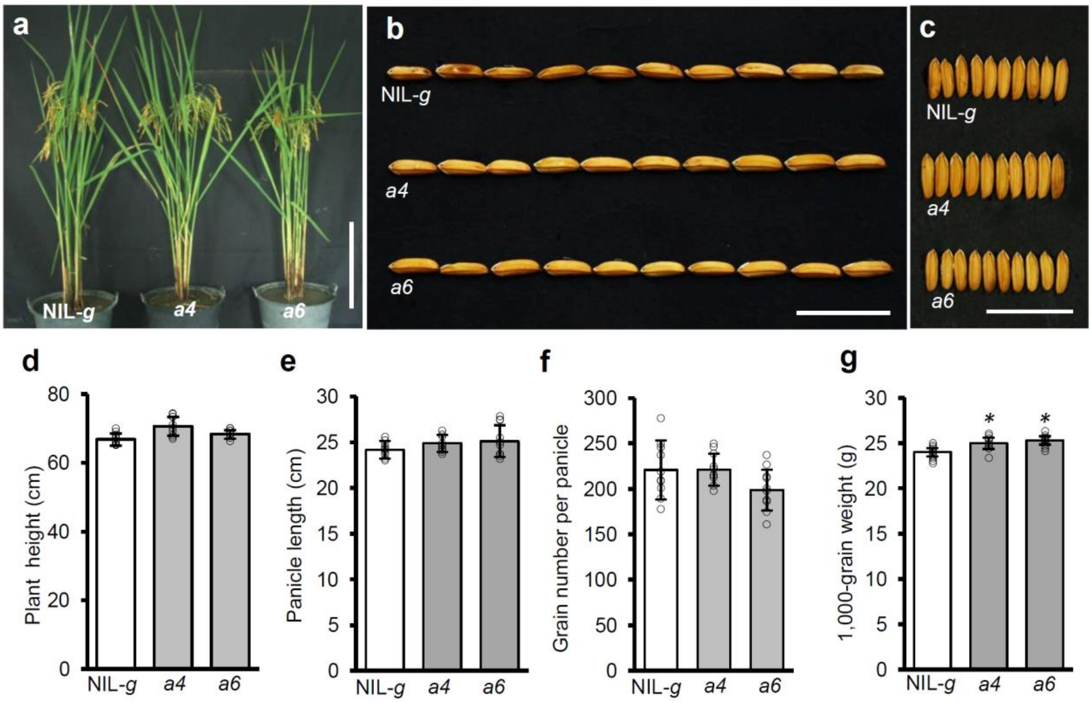


**Supplementary Figure 4**. **Agronomic traits in the *s1a4* and *s1a6* mutants.** (**a**) The architecture of the *s1a4* (*a4*) and *s1a6* (*a6*) mutants. Bar = 30 cm. (**b, c**) Yield-related traits of the *a4* and *a6* mutants. Bars = 2 cm. (**b**) Grain length. (**c**) Grain width. (**d–g**) Statistical analyses of the plant height (**d**), panicle length (**e**), grain number per panicle (**f**), and 1000-grain weight (**g**) of the *a4* and *a6* mutants. Data are mean ± SD ($n$ = 10). *, statistical significance at $P < 0.05$ in Student's $t$-test. Source data of Supplementary Figure 4d-4g are provided as a Source Data file.

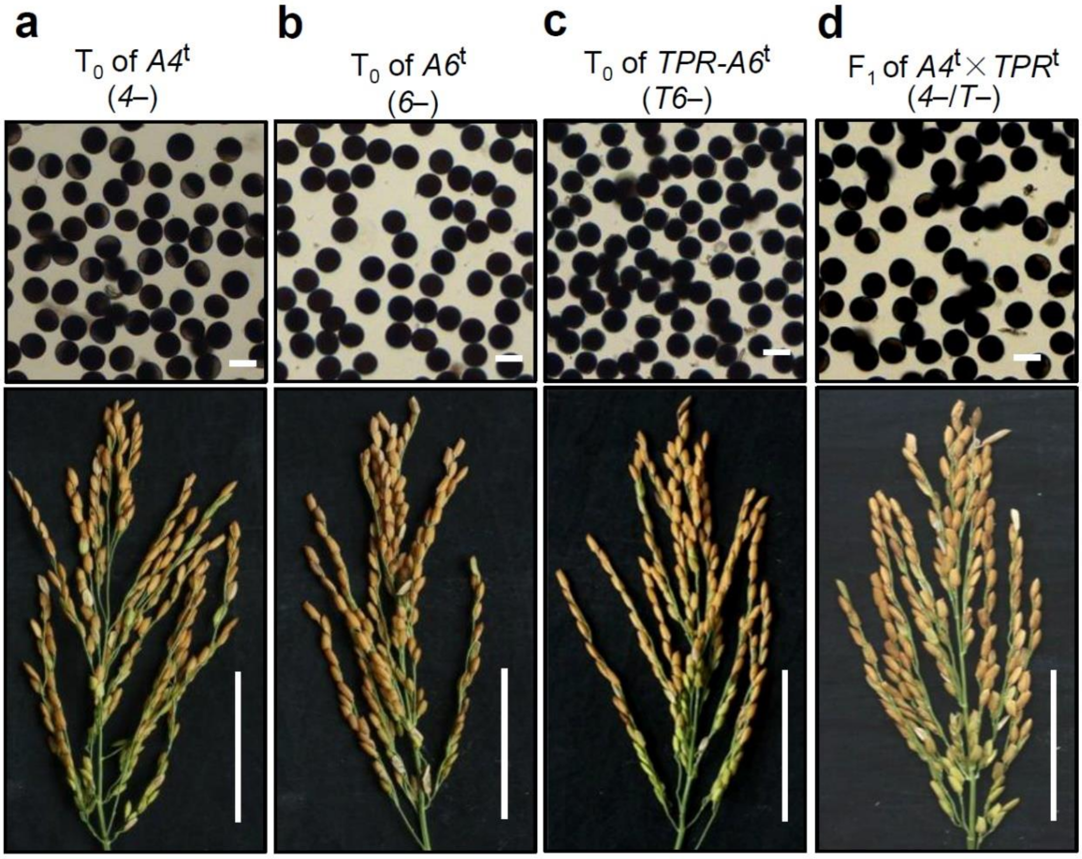


**Supplementary Figure 5**. **Phenotype of the transgenic plants containing one or two *S1A4,* *S1A6,* and *S1TPR* transgenes.** None of the *S1A4*$^{t}$ (*A4*$^{t}$), *S1A6*$^{t}$ (*A6*$^{t}$), *S1TPR-S1A6*$^{t}$ (*TPR-A6*$^{t}$), or $F_1$ (*A4*$^{t}$ × *TPR*$^{t}$) plants, in a hemizygous state, had sterile pollen (top) or spikelets (bottom). Bars represent 50 μm for pollen and 5 cm for panicles.

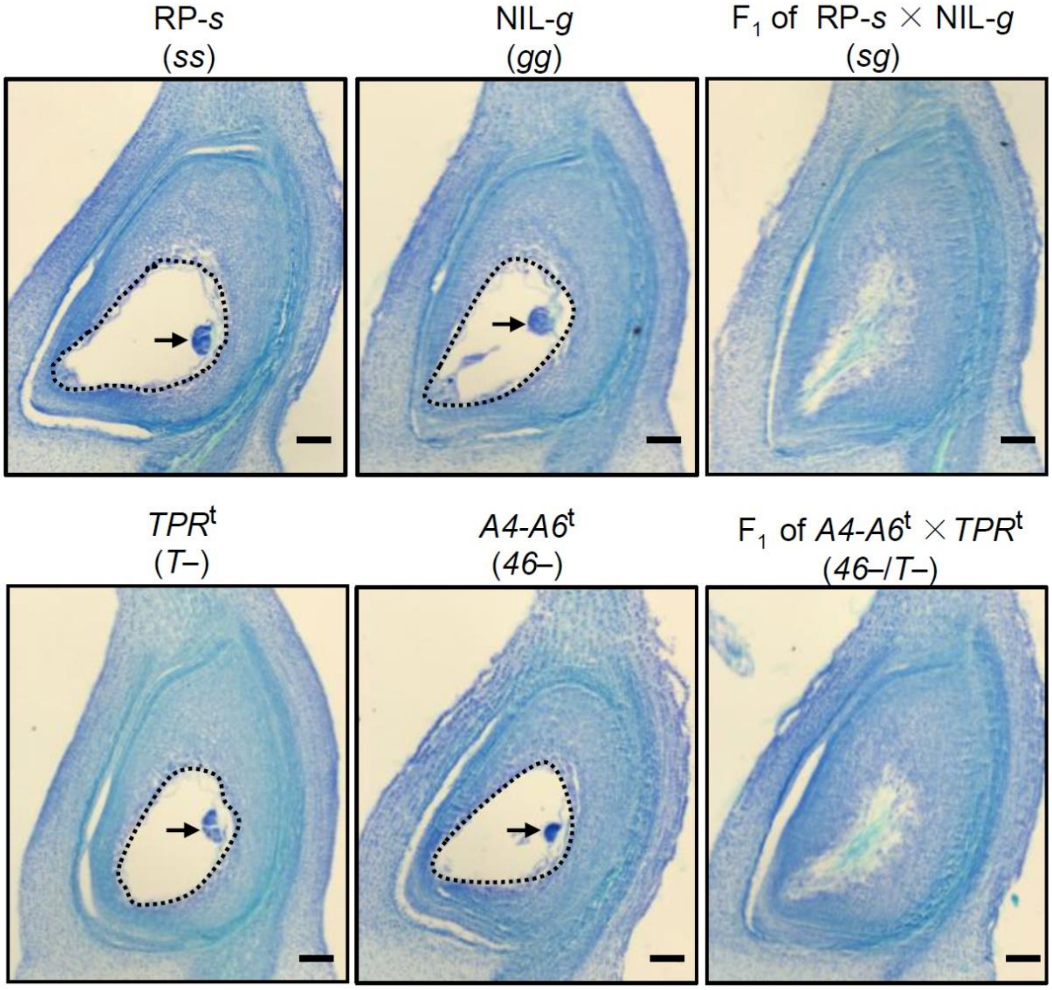


**Supplementary Figure 6**. **Longitudinal section of embryo sacs from different genotypes**. The embryo sacs of $F_1$ plants derived from RP-*s* × NIL-*g* or *S1A4*-*S1A6*$^{t}$ × *S1TPR*$^{t}$ (*A4-A6*$^{t}$ × *TPR*$^{t}$) are abnormal. The dashed frames indicate the embryo sac and the arrows indicate the antipodals. Scale bars = 50 µm.

**a**

```
   1 gcggccaccc ccaaccctaa aacatttccc acccaccgcg ccgccgcctt ctcgagtcgt
  61 ctcctctact tctcgacgtg cgccggctcg acctcgcctc ctccgccgcc ggccgccgta
 121 ctctcgcctt ctccctcgtt tccggtgagt cgatctgctg ctgaggtcgt cttctccggc
 181 tccggtctcc ggttgcgcag gtactaacct tttcacccta acggcctaac ctaaccctgg
 241 atctacatct cgatctcgtt ctgatgcgtg tgctgtgctc cctcccttct tgttcatata
 301 ggtctacctc gagcgggtgc ggcgcggcct ccttcctctc ctcctcctcc ggcttaaggc
 361 acgttgaccc ttgtcccccct ctcctcctcc tccgccgcta gatcgatcgg ttctgtggtc
 421 gctcgctctg atggattgaa tcgaaggtta ggggtccatg cggtgaagta gtcctttgga
 481 tccgcattgt ttctattcgt agatggtgga agtagtcctt tttttttttt gactaactca
 541 ctggaagaga aaagatgttt ccaaacatca atagaagtag tcttttttat ctgtgccatt
 601 tctgttctga tatgcttgcc atgcttgtgc gatctgtttg tggcttaggt ggttgatctg
 661 gagttgtagg gttgtgcttg tagtctgctc ttagatgagt tcttctcatg atctgctggg
 721 tcttttggtt agggttaagc tagatttgct cgtgatttta gattcgtctt tgtatatggt
 781 acccttgtcc atttatgatg ctgctgtctg ggtttatgcg tttgatttgt ccaaccgatt
 841 ttgtaagcat gtgctttacg tttgataggt ctgattgagt tgttgctgta cattggagta
 901 gcataatatc ttctcaacta gtagctcttg gccttaacct tttcttttat gatactcttg
 961 gccttaattt gaatctgcat gtgtaatcca atataaataa tgtttccagg aaaatggcat
1021 cgaacataca atctgaagct gagtcagctc gtgatagagc ccaagaatcg gtggtgattg
1081 ttcgagtcaa tacagatcct aatgagtact gttgtggttg cgtagtcagg tccaagtttg
1141 ttgggggatc cggaaacaga accacactgg ttataacttc atctaagttt gtacagggtc
1201 gagagaacga tttgacggtt gttttctgga acaaaaaaga gttaaaggct acctttctta
1261 ggacacatgg tgcgttttgt ttactggcta ctgatttcta cctttggtgc cagcctattc
1321 acttgttgga aggcaacgct gggctggaga attcgcgcac gttcatgcga gttccgctca
1381 accatagtac aacccggttt gtgttcactt ataccagtag taggtcggtt gagtcgtacc
1441 cagttgaaac tccaaatcat gcagtaccaa attcacatga atacttcatg gtcagctgta
1501 gctattttca aaagaccagc aaaggagtca gcagattaac aggtgcccct gtattttgta
1561 cggggggatgc tgggacagct ggaaggacca ttggtatcat tttgcaggat tgtcgccctg
1621 caacaggttg ttcaggtgct gaatttaaag ttgcactcaa tgcaagccat ctccagaagg
1681 tgctatcgat tcttgatcca ccagatccac cacaaaagag gaaccacaac cttagcgggg
1741 gtaagaagag gaaggctaca ggaagtgggg gaggaagagg aaggcggcaa agagtctagg
1801 ttgttgttag agaaaagttc tgatgagagg tggggtgaca agaatgtctg tcattttgtt
1861 gctgtatccc aactattaca attcatctct tagtctataa tgcactatgt atccgttcta
1921 tcattataac ttatcaattt gcgggcttag tctttaatgc caaa
```

**b**

```
   1 MASNIQSEAE SARDRAQESV VIVRVNTDPN EYCCGCVVRS KFVGGSGNRT TLVITSSKFV
  61 QGRENDLTVV FWNKKELKAT FLRTHGAFCL LATDFYLWCQ PIHLLEGNAG LENSRTFMRV
 121 PLNHSTTRFV FTYTSSRSVE SYPVETPNHA VPNSHEYFMV SCSYFQKTSK GVSRLTGAPV
 181 FCTGDAGTAG RTIGIILQDC RPATGCSGAE FKVALNASHL QKVLSILDPP DPPQKRNHNL
 241 SGGKKRKATG SGGGRGRRQR V.
```

**Supplementary Figure 7**. **Nucleotide and amino acid sequences of *S1A4*.** (**a**) The transcript of *S1A4* (*A4*) has three exons (red), with the coding sequence located in the third exon. The start (ATG) and stop (TAG) codons are marked in blue. (**b**) The deduced protein sequence of A4.

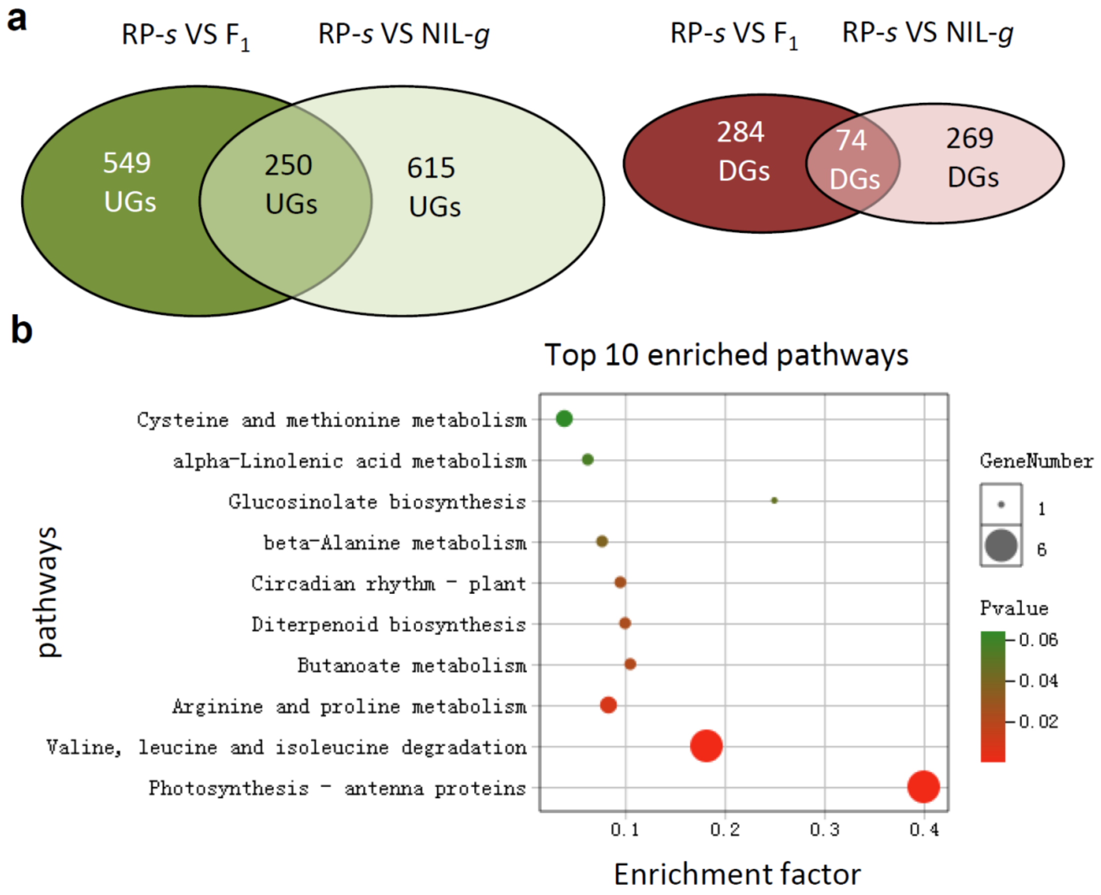


**Supplementary Figure 8. Transcriptome analysis of anthers from different genotypes. (a)** Venn diagram showing the upregulated genes (UGs) and downregulated genes (DGs) in the anthers of $F_1$ and NIL-*g* in comparison with RP-*s*, including those commonly up- or downregulated in both genotypes. The anthers were analyzed between the microspore mother cell stage and the meiosis stage. (**b**) The common UGs and DGs were annotated using the KEGG pathway database. Six genes involved in branched-chain amino acid (BCAA; i.e., valine, leucine, and isoleucine) degradation and six genes involved in photosynthesis were significantly upregulated in the anthers of $F_1$ and NIL-*g* relative to RP-*s*. Source data are provided as a Source Data file.

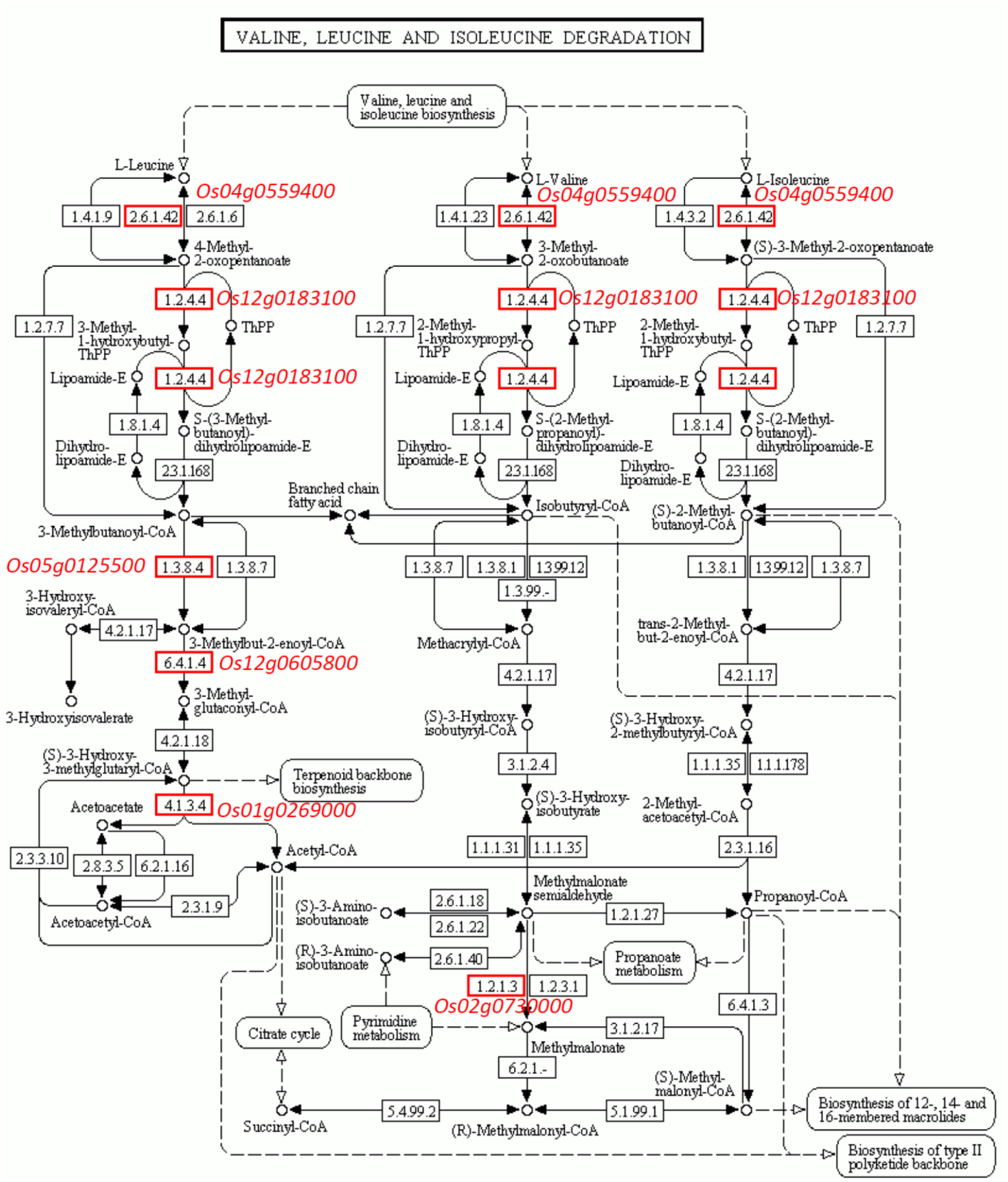


**Supplementary Figure 9. Degradation pathway of valine, leucine, and isoleucine (branched-chain amino acids, BCAAs) in the KEGG database.** The upregulated genes involved in the BCAA degradation pathway are marked in red.

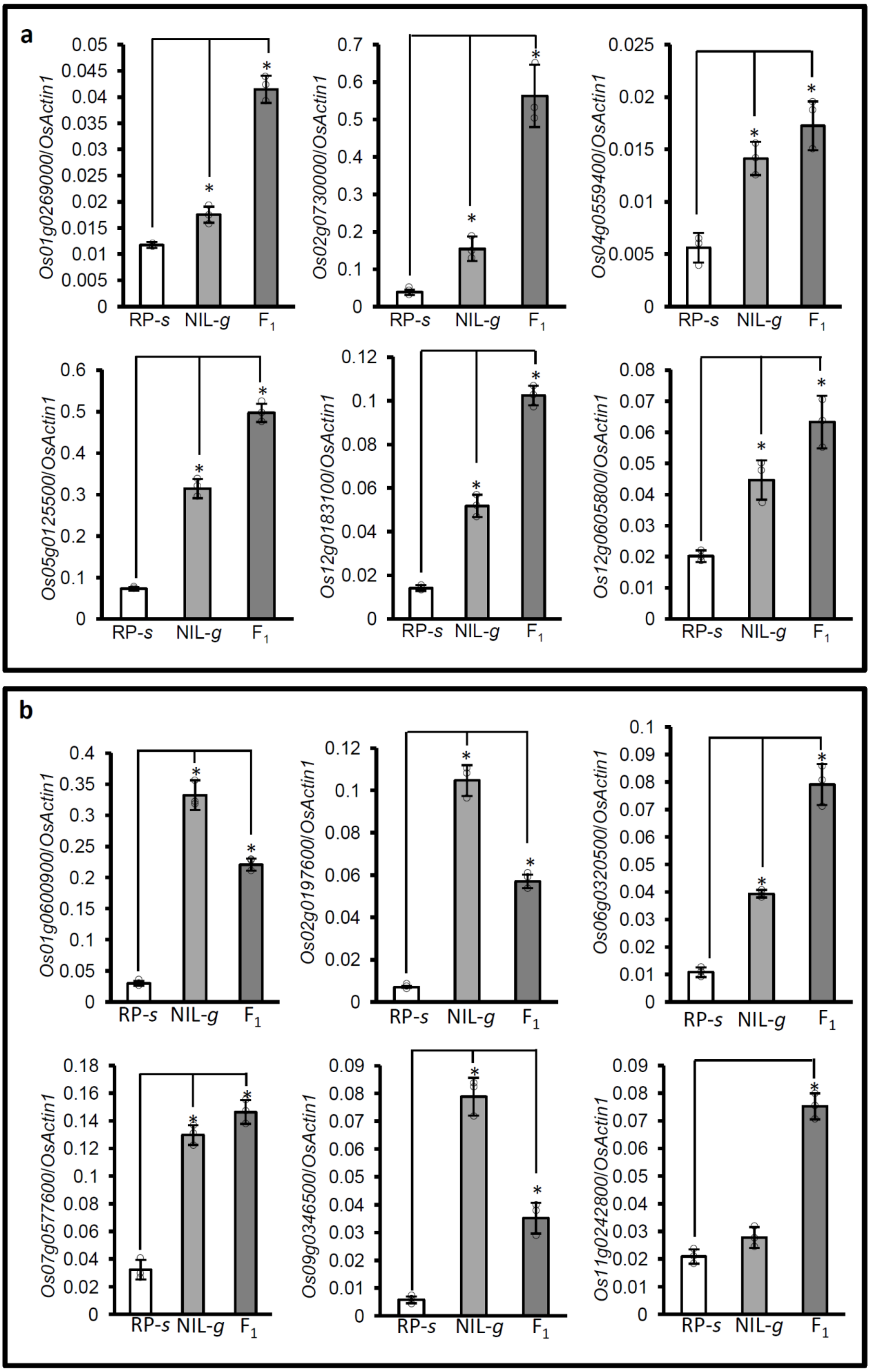


**Supplementary Figure 10. Validation of differentially expressed genes using qRT-PCR.** Expression analysis of the genes involved in branched-chain amino acid

(BCAA) degradation (**a**) and the photosynthesis (**b**) pathways that were upregulated in the anthers of NIL-*g* and $F_1$ relative to RP-*s*. The anthers were analyzed between the microspore mother cell stage and the meiosis stage. *OsActin1* serves as an internal reference gene. Data are mean ± SD ($n$ = 3). *, statistical significance at $P < 0.05$ in Student's *t*-test. Source data of Supplementary Figure 10 are provided as a Source Data file.

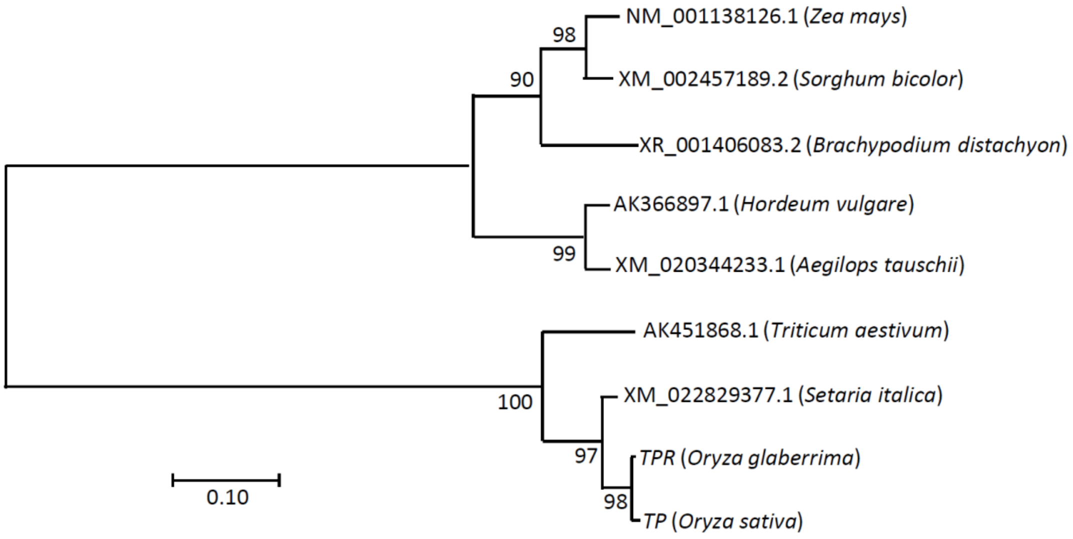


**Supplementary Figure 11. Phylogenetic tree of the putative *S1* locus *S1TPR* (*TPR*) orthologs in the Poaceae.** The putative orthologs were identified by searching the Poaceae sequences in the NCBI database using the nucleotide coding sequence of *S1TPR* to construct a phylogenetic tree. The bootstrap values indicate the percentage of support from 1,000 sampled trees. The scale bar indicates the number of nucleotide substitutions per site.

a

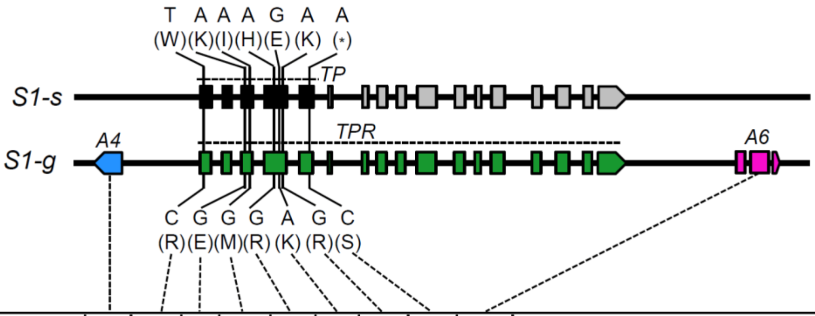


b

| Haplotype | Allele variant | A4 | 1 | 2 | 3 | 4 | 5 | 6 | 7 | A6 | Species (number of accessions) |
|---|---|---|---|---|---|---|---|---|---|---|---|
| *TPR* | Ancestral Allele 1 | - | C | A | G | G | G | G | C | - | *S. italica* (2) *O. officinalis* (6) *O. minuta* (4) *O. longsistaminata* (11), *O. meridionalis* (1) |
| *TPR*/*TP* | Alleles 1/1-1 | - | C | A | G | G | G | G | C/A | - | *O. officinalis* (1) *O. eichingeri* (2) *O. rhizomatis* (1) *O. longsistaminata* (9), *O. meridionalis* (1) |
| *TP* | Allele 1-1 | - | C | A | G | G | G | G | A | - | *O. rhizomatis* (1) *O. longsistaminata* (4), *O. meridionalis* (1) |
| | Allele 1-2 | - | C | A | A | G | G | G | A | - | *O. meridionalis* (3) |
| | Allele 1-3 | - | C | A | A | G | G | A | A | - | *O. meridionalis* (4), *O. rufipogon* (51), , *O. sativa* (64) |
| | Allele 1-4 | - | C/T | A | A | G | G | A | A | - | *O. rufipogon* (3) |
| | Allele 1-5 | - | C/T | A | A | G/A | G | A | A | - | *O. rufipogon* (3) |
| | Allele 1-6 | - | C/T | A | A | A | G | A | A | - | *O. rufipogon* (7) |
| | Allele 1-7 | - | C | A | A | A | G | A | A | - | *O. rufipogon* (1) |
| | Allele 1-8 | - | T | A | A | A | G | A | A | - | *O. rufipogon* (3), *O. sativa* (52) |
| *TP* with *A4* or/& *A6* | Allele 1-9 | + | C | A | G | G | G | G | A | - | *O. meridionalis* (2) |
| | Allele 1-10 | + | C/T | A | G | G | G | G | A | - | *O. meridionalis* (4) |
| | Allele 1-11 | + | C | A | G | G | G | A | A | - | *O. meridionalis* (1) |
| | Allele 1-12 | - | C | A | A | G | G | G | A | + | *O. meridionalis* (1) |
| | Allele 1-13 | - | C | A | G | G | G | A | A | + | *O. meridionalis* (4) |
| | Allele 1-14 | + | C | A | G/A | G | G | G/A | A | + | *O. meridionalis* (1) |
| | Allele 1-15 | + | T | A | A | G | G | A | A | + | *O. meridionalis* (1) |
| *TPR* with *A4* & *A6* | Allele 1-16 | + | C | A | G | G | G | G | C | + | *O. meridionalis* (13) |
| *TPR* | Allele 2-1 | - | C | A | G | G | A | G | C | - | *O. barthii* (2) |
| *TPR* with *A4* or/& *A6* | Allele 2-2 | + | C | G | G | G | A | G | C | - | *O. barthii* (10) |
| | Allele 2-3 | - | C | G | G | G | A | G | C | + | *O. barthii* (1) |
| | Allele 2-4 | + | C | G | G | G | A | G | C | + | *O. barthii* (52), *O. glaberrima* (133) |

**Supplementary Figure 12. Gene structures of the *S1* alleles and the haplotypes and allele variations in the *Oryza* species.** + and - indicate the presence and absence, respectively, of the genes *S1A4* (*A4*) and *S1A6* (*A6*). * in *S1TP* (*TP*) indicates the premature stop codon caused by the C-to-A mutation. The numbers of accessions carrying the alleles are indicated in parentheses.

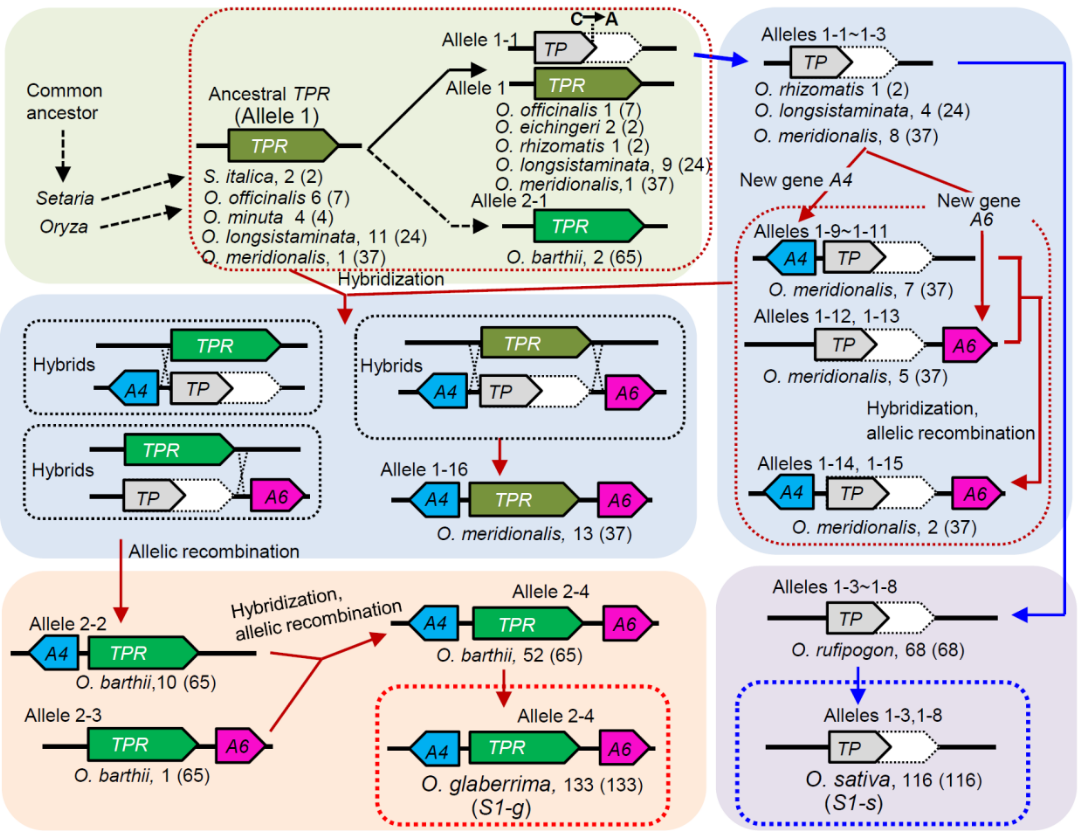


**Supplementary Figure 13**. **Evolution of the *S1* allele variants in *Oryza*.** An ancestral primitive *S1TPR* (*TPR*) structure (Allele 1), which lacked the flanking genes *S1A4* (*A4*) and *S1A6* (*A6*), was detected in both analyzed accessions of an outgroup species, *Setaria italica*, and some accessions of *Oryza* wild species (the total number of investigated accession is in parentheses). The primary *S1TP* (*TP*) (Allele 1-1) was detected in the heterozygous state (Allele 1/Allele 1-1) in some accessions of wild *Oryza* species. In one lineage, some of the resultant *TP* alleles (Alleles 1-1 to 1-16) passed through a bottleneck and migrated into *O. rufipogon*, with two (Alleles 1-3 and 1-8) eventually being fixed as the current *S1-s* alleles in *O. sativa*. In another lineage, the new genes *A6* and *A4* arose upstream and downstream of *TP*, respectively, to generate the *A4-TP* and *TP-A6* structures. The new structure *A4-TP-A6* was likely eventually generated by recombination between *A4-TP* and *TP-A4* during natural hybridization among the ancestral wild species. The three-gene structure was generated in *O. barthii*, and further migrated into *O. glaberrima* where it formed the functional *S1-g* allele.

**Supplementary Table 1. CRISPR/Cas9-edited *a2~a6* mutants in *S1-g*.**

| Lines | Edited targets |
|---|---|
| *A2*-WT | GAGGCTACGATGCGGGCAGTCGG |
| *a2-1* | GAGGCTACGATGC**-168 bp DEL**-GGG |
| *a2-2* | GAGGCTACGATGCGGGC**T**AGTCGG |
| *A3*-WT | AATAATCATCTCGGTGCAACTGG |
| *a3-1* | AATAATCATCTCGGTGC**-**ACTGG |
| *a3-2* | AATAATCATCTCGGTGCAA**AA**CTGG |
| *A4*-WT | TGATAGAGCCCAAGAATCGGTGG |
| *a4-1* | TGATAGAGCCCAAGAAT**T**CGGTGG |
| *a4-2* | TGATAGAGCCCAAGAAT**-**GGTGG |
| *A5*-WT | CAATTGCAGGGCGTACGATCTGG |
| *a5-1* | CAATTGCAGGGCGTACGA**A**TCTGG |
| *a5-2* | CAATTGCAGGGCGTAC**-**ATCTGG |
| *A6*-WT | AGACGAGCGCTGAGCTCGCAAGG |
| *a6-1* | AGACGA**-19 bp DEL-**CGCGGTGCTCA |
| *a6-2* | AGACGAGCGCTGAGCTC**T**GCAAGG |

Notes: mutations in the *s1a2~s1a6* (*a2~a6*) alleles are shown in bold. The PAM sequences are underlined.

**Supplementary Table 2. Segregation analysis of the *S1* locus in the $mF_2$ populations derived from the cross RP-*s* × *a2~a6*.**

| $mF_2$ family | *S1* locus genotype | No. of plants | Pollen fertility | Spikelet fertility | $\chi^2$ (1:2:1) |
|---|---|---|---|---|---|
| RP-*s* × *a2-1* | $g^mg^m$ | 146 | FF | FF | 348.84*** |
| | $g^ms$ | 21 | SS | SS | |
| | *ss* | 0 | - | - | |
| RP-*s* × *a2-2* | $g^mg^m$ | 154 | FF | FF | 364.93*** |
| | $g^ms$ | 23 | SS | SS | |
| | *ss* | 0 | - | - | |
| RP-*s* × *a3-1* | $g^mg^m$ | 138 | FF | FF | 281.49*** |
| | $g^ms$ | 35 | SS | SS | |
| | *ss* | 0 | - | - | |
| RP-*s* × *a3-2* | $g^mg^m$ | 130 | FF | FF | 249.00*** |
| | $g^ms$ | 39 | SS | SS | |
| | *ss* | 0 | - | - | |
| RP-*s* × *a4-1* | $g^mg^m$ | 45 | FF | FF | 0.10 |
| | $g^ms$ | 93 | FF | FF | |
| | *ss* | 48 | FF | FF | |
| RP-*s* × *a4-2* | $g^mg^m$ | 47 | FF | FF | 0.43 |
| | $g^ms$ | 97 | FF | FF | |
| | *ss* | 43 | FF | FF | |
| RP-*s* × *a5-1* | $g^mg^m$ | 122 | FF | FF | 297.13*** |
| | $g^ms$ | 16 | SS | SS | |
| | *ss* | 0 | - | - | |
| RP-*s* × *a5-2* | $g^mg^m$ | 121 | FF | FF | 286.99*** |
| | $g^ms$ | 18 | SS | SS | |
| | *ss* | 0 | - | - | |
| RP-*s* × *a6-1* | $g^mg^m$ | 49 | FF | FF | 0.19 |
| | $g^ms$ | 103 | FF | FF | |
| | *ss* | 48 | FF | FF | |
| RP-*s* × *a6-2* | $g^mg^m$ | 55 | FF | FF | 1.29 |
| | $g^ms$ | 130 | FF | FF | |
| | *ss* | 66 | FF | FF | |

Notes: The *S1*-$g^m$ ($g^m$) allele contained a mutated gene *s1a2~s1a6* (*a2~a6*) in *S1-g*. ***, significance at $P < 0.001$ in the Chi-square test. Two independent knockout $mF_2$ families were analyzed for each gene, and the underlined crosses are shown in Supplementary Fig. 3. Source data are provided as a Source Data file.

**Supplementary Table 3. Segregation analysis of *TPR*$^t$ in the $T_1$ generation.**

| $T_1$ line | *S1* genotype | *TPR*$^t$ genotype | No. of plants | Pollen fertility | Spikelet fertility | $\chi^2$ (1:2:1) |
|---|---|---|---|---|---|---|
| *TPR*$^t$#13 | *ss* | *TT* | 65 | FF | FF | 0.29 |
| | | *T*– | 120 | FF | FF | |
| | | –– | 63 | FF | FF | |
| *TPR*$^t$#18 | *ss* | *TT* | 59 | FF | FF | 1.43 |
| | | *T*– | 142 | FF | FF | |
| | | –– | 67 | FF | FF | |

Notes: $\chi^2$ (1:2:1) test was performed for the segregation of the transgene *S1TPR*$^t$ (*TPR*$^t$). *T* and –, presence and absence of the transgene, respectively; FF, fully fertile (> 90%). Two independent transgenic lines were analyzed, and the segregation data of *TPR*$^t$#13 are shown in Fig. 1e. Source data are provided as a Source Data file.

**Supplementary Table 4. Segregation analysis of *A4-A6*$^{t}$ in the $T_1$ generation.**

| $T_1$ generation | *S1* locus genotype | *A4-A6*$^{t}$ genotype | No. of plants | Pollen fertility | Spikelet fertility | $\chi^2$ (1:2:1) |
|---|---|---|---|---|---|---|
| *A4-A6*$^{t}$#19 | *ss* | *4646* | 48 | FF | FF | 0.18 |
| | | *46–* | 97 | FF | FF | |
| | | –– | 45 | FF | FF | |
| *A4-A6*$^{t}$#11 | *ss* | *4646* | 54 | FF | FF | 0.75 |
| | | *46–* | 105 | FF | FF | |
| | | –– | 46 | FF | FF | |

Note: Two independent transgenic lines were analyzed, and the segregation data of *S1A4-S1A6*$^{t}$#19 (*A4-A6*$^{t}$#19) are shown in Fig. 1e. Source data are provided as a Source Data file.

**Supplementary Table 5. The segregation analysis of *TPR* in $F_2$ populations derived from the cross *A4-A6*$^t$ × *TPR*$^t$.**

| $F_2$ family | *A4-A6*$^t$ genotype | *TPR*$^t$ genotype | No. of plants | Pollen fertility | Spikelet fertility | $\chi^2_{(46)}$ (1:2:1) | $\chi^2_{(T)}$ (1:2:1) |
|---|---|---|---|---|---|---|---|
| *A4-A6*$^t$#19 × *TPR*$^t$#13 | *4646* | *TT* | 55 | FF | FF | 0.20 | 397.73*** |
| | | *T–* | 16 | SS | SS | | |
| | | –– | 0 | - | - | | |
| | *46–* | *TT* | 106 | FF | FF | | |
| | | *T–* | 37 | SS | SS | | |
| | | –– | 0 | FF | FF | | |
| | –– | *TT* | 52 | FF | FF | | |
| | | *T-* | 15 | FF | FF | | |
| | | –– | 0 | - | - | | |
| *A4-A6*$^t$#11 × *TPR*$^t$#18 | *4646* | *TT* | 41 | FF | FF | 0.85 | 287.08*** |
| | | *T–* | 11 | SS | SS | | |
| | | -- | 0 | - | - | | |
| | *46–* | *TT* | 72 | FF | FF | | |
| | | *T–* | 24 | SS | SS | | |
| | | –– | 0 | FF | FF | | |
| | –– | *TT* | 35 | FF | FF | | |
| | | *T–* | 8 | FF | FF | | |
| | | –– | 0 | - | - | | |

Note: The segregation data of *S1A4-S1A6*$^t$#19 × *S1TPR*$^t$#13 (*A4-A6*$^t$#19 × *TPR*$^t$#13) are shown in Fig. 1e. ***, significance at $P < 0.001$ in the Chi-square test. Source data are provided as a Source Data file.

**Supplementary Table 6. Segregation analysis of *S1* and *TPR*$^{t}$ in $F_2$ populations derived from the cross *TPR*$^{t}$ × NIL-*g*.**

| $F_2$ Population | *S1* genotype | *TPR*$^{t}$ genotype | No. of plants | Pollen fertility | Spikelet fertility | $\chi^2_{(S1)}$ (4:4:1) | $\chi^2_{(T)}$ (4:4:1) |
|---|---|---|---|---|---|---|---|
| *TPR*$^{t}$#13 × NIL-*g* | *gg* | *TT* | 36 | FF | FF | 1.05 | 0.13 |
| | | *T*– | 60 | FF | FF | | |
| | | –– | 30 | FF | FF | | |
| | *gs* | *TT* | 61 | FF | FF | | |
| | | *T*– | 68 | PF | PF | | |
| | | –– | 5 | SS | SS | | |
| | *ss* | *TT* | 35 | FF | FF | | |
| | | *T*– | 3 | FF | FF | | |
| | | –– | 0 | - | - | | |
| *TPR*$^{t}$#18 × NIL-*g* | *gg* | *TT* | 19 | FF | FF | 1.62 | 0.82 |
| | | *T*– | 45 | FF | FF | | |
| | | –– | 22 | FF | FF | | |
| | *gs* | *TT* | 40 | FF | FF | | |
| | | *T*– | 43 | PF | PF | | |
| | | –– | 4 | SS | SS | | |
| | *ss* | *TT* | 26 | FF | FF | | |
| | | *T*– | 2 | FF | FF | | |
| | | –– | 0 | - | - | | |

Notes: FF, fully fertile (> 90%); PF, partially fertile (70~75%); SS, semi-sterile (45~55%). The segregation data of *S1TPR*$^{t}$#13 × NIL-*g* (*TPR*$^{t}$#13 × NIL-*g*) are shown in Fig. 2. Source data are provided as a Source Data file.

**Supplementary Table 7. Segregation analysis of *S1* and *TPR*$^{t}$ in $F_2$ populations derived from the cross *TPR*$^{t}$ × *tpr*.**

| $F_2$ Population | *S1* genotype | *TPR*$^{t}$ genotype | No. of plants | Pollen fertility | Spikelet fertility | $\chi^2_{(S1)}$ (1:2:1) | $\chi^2_{(T)}$ (1:2:1) |
|---|---|---|---|---|---|---|---|
| *TPR*$^{t}$#13 × *tpr* | $g^m g^m$ | *TT* | 60 | FF | FF | 0.07 | 615.17*** |
| | | *T*– | 7 | SS | SS | | |
| | | –– | 0 | - | - | | |
| | $g^m s$ | *TT* | 123 | FF | FF | | |
| | | *T*– | 16 | SS | SS | | |
| | | –– | 0 | FF | FF | | |
| | *ss* | *TT* | 63 | FF | FF | | |
| | | *T*– | 5 | FF | FF | | |
| | | –– | 0 | - | - | | |
| *TPR*$^{t}$#18 × *tpr* | $g^m g^m$ | *TT* | 46 | FF | FF | 0.50 | 494.59*** |
| | | *T*– | 6 | SS | SS | | |
| | | –– | 0 | - | - | | |
| | $g^m s$ | *TT* | 106 | FF | FF | | |
| | | *T*– | 11 | SS | SS | | |
| | | –– | 0 | FF | FF | | |
| | *ss* | *TT* | 49 | FF | FF | | |
| | | *T*– | 8 | FF | FF | | |
| | | –– | 0 | - | - | | |

Notes: FF, fully fertile (> 90%); SS, semi-sterile (45~55%). The segregation data of *S1TPR*$^{t}$#13 × *s1tpr* (*TPR*$^{t}$#13_× *tpr*) are given in Fig. 3e. ***, significance at $P < 0.001$ in the Chi-square test. Source data are provided as a Source Data file.

## Supplementary Table 8. Primers used in this study.

| Primer Name | Sequence (5′ – 3′) | Purpose |
|---|---|---|
| TPR-linkF | CAGCTATGACCATGATTACGAATTCGCTTGTTCCCATGGATAGCATGAGC | Transgenic constructs for complementation test |
| TPR-linkR | CTGCAGGTCGACTCTAGAGGATCCGACATTGGAGGCAAGCTCC | |
| TPR-A6-linkF | GGAGCTTGCCTCCAATGTCGGAGGATTACGAGGATGATCTG | |
| TPR-A6-linkR | CTGCAGGTCGACTCTAGAGGTCACTCCAACGTGATCAGC | |
| A4-linkF | CAGCTATGACCATGATTACGAATTCGATAGAGCGGCGGAAGATACGAG | |
| A4-linkR | CTGCAGGTCGACTCTAGAGACGCGTCTCCCTAAGCTCGCGAGCTTTGTC | |
| A6-linkF | CAGCTATGACCATGATTACGAATTCACATGTCAGCTGCCACGTCAAC | |
| A6-linkR | CTGCAGGTCGACTCTAGAGGATCCTGATCTCCTACCTTGCACTC | |
| A4-A6-linkF | GACAAAGCTCGCGAGCTTAGGGAGACGGGAGGATTACGAGGATGATCTG | |
| A4-A6-linkR | CTGCAGGTCGACTCTAGAGAGTCACTCCAACGTGATCAGC | |
| a2F | GCCGGAGGCTACGATGCGGGCAGT | Knockout editing |
| a2R | AAACACTGCCCGCATCGTAGCCTC | |
| a3F | GCCGAATAATCATCTCGGTGCAAC | |
| a3R | AAACGTTGCACCGAGATGATTATT | |
| a4F | GCCGTGATAGAGCCCAAGAATCGG | |
| a4R | AAACCCGATTCTTGGGCTCTATCA | |
| a5F | GCCGCAATTGCAGGGCGTACGATC | |
| a5R | AAACGATCGTACGCCCTGCAATTG | |
| a6F | GCCGAGACGAGCGCTGAGCTCGCA | |
| a6R | AAACTGCGAGCTCAGCGCTCGTCT | |
| LBF | TTCGATGATGCAGCTTGGGCGCAG | Segregation analysis |
| TPR$^{t}$#13-P1 | GAGATGCCATCCTATGTAAC | |
| TPR$^{t}$#13-P2 | CTGTGCTGATTGACCAATCACTG | |
| TPR$^{t}$#18-P1 | GCACTGGCTATCACCTGTG | |
| TPR$^{t}$#18-P2 | GGAAGTGAATTATTGATCACTC | |
| A4-A6$^{t}$#11-P1 | GACGGTCAAACATTTAGACAG | |
| A4-A6$^{t}$#11-P2 | GGTGTGGCAAGTATGGTATC | |
| A4-A6$^{t}$#19-P1 | CTTCCCAGCGTGTGTATTATG | |
| A4-A6$^{t}$#19-P2 | GATCGGGTGTTGCGTACTTG | |
| 2170F | GGACCTTTCTTAGGTTCTATTTAG | |
| 2170R | CTATAGCTTCTTAGGATTTGTAGC | |
| RTActinF | ACCTCATGAAGATCCTGACG | Analysis of gene expression using RT-PCR |
| RTActinR | ACAGATAGGCCGGTTGAAAA | |
| RTA1F | ATGTCGAACAAGGCTACGAATTC | |
| RTA1R | CTAGTTCGATTTTGTGAGAGT | |
| RTA2F | GCAATTCTCTTTCTTCATCAAC | |
| RTA2R | CCACCGAGGACGCGGAAAC | |
| RTA3F | GGATGATTGGAATATTTTACATAGG | |
| RTA3R | GGTCAGGAAATTTTCCTCTC | |
| RTA4F | ATGGCATCGAACATACAATCTG | |
| RTA4R | CTAGACTCTTTGCCGCCTTCC | |
| RTA5F | CAATTTCAGCTTGGAGGATTACGA | |
| RTA5R | GCGATGCCTCTTAACTATAC | |
| RTA6F | ATGTCGAAGACAAATCCCAAC | |
| RTA6R | TCAATGCAGCGCATCAAGGAC | |
| qActinF | ACCACAGGTAGCAATAGGTA | Analysis of gene expression using quantitative RT-PCR |
| qActinR | CACATTCCAGCAGATGTGGA | |
| qTPRF | GAGATCTGCAAGATTCTAGC | |
| qTPRR | CTGTATTGAAGCTGGCCAGTA | |
| qA2F | CATCCTGCAAATAATTACACG | |
| qA2R | GGACACAGCTGTCAAACCTG | |
| qA3F | GGATGATTGGAATATTTTACATAGG | |
| qA3R | GCAGATCCAAGGCAGCAAAG | |
| qA4F | TCTCCGGTTGCGCAAGGTCT | |
| qA4R | GGATCCCCCAACAAACTTGGAC | |
| qA5F | CAATTTCAGCTTGGAGGATTACGA | |
| qA5R | GCGATGCCTCTTAACTATAC | |
| qA6F | GGCAGCTTACCTTTGAACCATC | |

| | |
|---|---|
| qA6R | CCAACATATTCAGTCGACACAAC |
| Os01g0269000F | CACGATACCTACGGCCAATC |
| Os01g0269000R | CAGCCCGTTCAGCATGTACAC |
| Os02g0730000F | GACGGCGAGCAATTCAAGAAG |
| Os02g0730000R | CCTCCACCGTGCTGAACTTG |
| Os04g0559400F | TGGGATGGACTGTGCAACTG |
| Os04g0559400R | CACTGGAAGTATGGTCAGTAAG |
| Os05g0125500F | GGACTGTGACAATGGTAAAG |
| Os05g0125500R | CACTAGTACCGGCTCCAATC |
| Os12g0183100F | GGTGTGATGAAGATGAATCTG |
| Os12g0183100R | CTCAGCAATCGCTCTTGCTC |
| Os12g0605800F | CCTGTCATGGTTATGGAAGC |
| Os12g0605800R | GTACAGGTCGTGAATCTCTG |
| Os01g0600900F | CATCGCCTTCAGCTAAGCTAG |
| Os01g0600900R | ACTACCGGTACAGATCTCAC |
| Os02g0197600F | GTACGTGCGTATGCGTATATG |
| Os02g0197600R | CCATGCGTGCAAATCAGTTG |
| OS06G0320500F | CAAGTTCGAGGAGTACAAGC |
| Os06g0320500R | GTTCTCGGGATGATGATGTC |
| Os07g0577600F | GAGGAGTTCTTCAGGTGTTG |
| Os07g0577600R | GTGCTCCATCCACAATTACATC |
| Os09g0346500F | CGTACGCCACCAACTTCGTC |
| Os09g0346500R | CATCATCTCGTCGCACTAAAC |
| Os11g0242800F | GAGTACTACCGGATCATCAAC |
| Os11g0242800R | CGTAGGCCTGGATGAAGAAC |
