# Supplementary material for: An asymmetric allelic interaction drives allele transmission bias in interspecific rice hybrids
Source: Nat Commun. 2019 Jun 7;10:2501. doi: 10.1038/s41467-019-10488-3 (PMC6555797; doi:10.1038/s41467-019-10488-3)
Supplement: Supplementary file 4 — Description of Additional Supplementary Files [file 41467_2019_10488_MOESM4_ESM.docx]

**Description of Additional Supplementary Files**

**File Name:** Supplementary Data 1
**Description:** This Dataset summarizes the distribution of S1 allele variants in Oryza species.
