# Supplementary material for: An asymmetric allelic interaction drives allele transmission bias in interspecific rice hybrids
Source: Nat Commun. 2019 Jun 7;10:2501. doi: 10.1038/s41467-019-10488-3 (PMC6555797; doi:10.1038/s41467-019-10488-3)
Supplement: Supplementary file 2 — Peer Review [file 41467_2019_10488_MOESM2_ESM.pdf]

Reviewers' comments:

Reviewer #1 (Remarks to the Author):

Reviewer 1#: The manuscript dealt with 'An asymmetric allelic interaction drives allele 1 transmission bias in interspecific rice hybrids". As well known, there is huge yield potential in the hybrid rice breeding. It is an important strategy for rice breeder to take advantage of this heterosis. However, hybrid sterility is a major form as reproductive isolation during domestication and hinders the exploitation of the heterosis displayed by inter-specific or inter-subspecific hybrid. In this manuscript, the authors demonstrated that three closely linked genes (S1A4, S1TPR and S1A6) in the African rice S1 allele constitute a gamete killer-protector system. Knockout of anyone of the three genes at this locus can overcome the interspecific reproductive barrier. Evolutionary analysis showed that the S1 loci arose from newly evolved genes and complex reorganization process. The manuscript would be of interest to a wide audience to understand the molecular mechanisms governing the incompatible interactions between the different species.
Here are some questions that need to be addressed by the authors.
1, The cloned hybrid sterility loci, such as S5, S7, Sa, Sc, S27/S28, are closely adjacent genetic interaction. Why is the asymmetric genetic interaction localized at the S1 locus? Whether do the interval genes, such as A5 and so on, play a role in the genetic interaction?

2, The S1 gene derived from African rice Oryza glaberrima, induced preferential abortion of both male and female gametes possessing its allelic alternative from Asian rice O. sativa only in the heterozygote. In this manuscript, the authors speculated that the co-existence of A4, TPR and A6 might produce a sterility signal in a sporophytic manner to kill gametes (both male and female), moreover, TPR expressed in the microspores and A6 (SSP) was not detected in EM and LM stage (Supplementary Fig. 1). It is better to provide more detailed data for expressions of the three genes in reproduction development?

3, The subcellular localization of these three proteins A4, A6 and TPR were localized in the nucleus, however, the authors showed that they produced a sterility signal in a sporophytic manner to kill gametes (both male and female). Is there any indirect result from these three gene interaction?

4, It is better to discuss more about the mechanism? For example, whether does the detrimental sterility signal need to be emitted by a complex of the three genes? Is there any other possible mechanisms underlying the detrimental sterility signal?

Reviewer #2 (Remarks to the Author):

This paper identified a killer-protector system at S1 locus responsible for hybrid sterility and segregation distortion between two cultivated rice species. This work substantially expanded our understanding of reproductive isolation. As shown by the work, the S1 locus is an important speciation locus as it affects both male and female sterility in the hybrids. The data obtained from genetic analyses are sound and convincing, revealing a complex interaction among the three genes at this locus that form a killer-protector system in regulating hybrid sterility. Although killer-protector system has been reported previously, a novel and interesting feature is the characterization of one causal gene, TPR, that kills the gamete in hybrids at sporophytic stage and is also responsible for rescuing the gamete in a gameotophytic manner. The subsequent sequence and divergence analysis describes an evolutionary picture with repect to the origin of such reproductive barrier. Together with other repored studies, the results indicate that the killer-protector system may be a general strategy for postzygotic reproductive isolation during the evolution. In addition, this result may serve the first step for a feasible approach to overcome the inter-specific reproductive isolation to faciliate the utilization of distant heterosis in hybrid rice breeding.

A few questions listed below might help imporve this work.
Major criticisms:
1 The interaction of A4 with A6 and TPR is very critical to the proposed working model. However, genetic interaction does not necessarily mean physical interaction of the proteins. The evidence based on the present data for interaction is quite weak. And a further downside is that the work has provided no clue as to how and on what the killer works and the possible cellular and biochemical processes leading to gamete abortion. This left with a feeling that we came to an abrupt end. Some additional work seems necessary for smoother ending.

2 As mentioned in the introduction, both male and female gametes carrying S1-s are selectively aborted. Thus how is the phenotype of the embryo-sac in the hybrid here? Based on Fig. 2b, the author analyzed the segregation ratio based on the assumption that the female gametes with s/- were aborted. Is there any experiment to support the assumption?
For Fig. 2a, I am wondering if ss/-- is not observed in the segregating population. If yes, to avoid ambiguity, ss/-- might be added in the genotype row with zero individual.
For Fig. 2c, the green, red, and blue colors are in accordance with the colors in Fig. 2b, which is quite clear. However, the white and grey colors have no correspondence in Fig, 2b, this might cause confusion.

3 I am wondering if the three components at S1 have orthologs in other grass species. The Result said that one ortholog of TPR was identified in foxtail millet. If the orthologs existed in other species, a phylogenetic tree might tell something about the origin and relationship of these genes. In addition, a haplotype network is recommended to trace the stepwise mutational step of different alleles. Is it possible that the most ancestral haplotype contains the A4-TPR-A6, and these genes were either lost or under rapid evolution in other linages? It is not clear to me (or is it clear yet?), based on the presently available resequencing data from all sources, whether there are any sativa accessions that have all three genes (and/or other of the A1 to A6 genes) albeit that they may not be functional. This may have important implication in the evolutionary study.

4. Since you have no clue at the moment on what and how the killer works, it may be premature to state "this S1 gamete killer-protector system is distinct from other Bateson-Dobzhansky-Muller type…". This is also related to the question in the previous paragraph about the evolutionary analysis.

Minor points:
1 Because of the complexity of the genes and alleles and interactions among the S1 locus, a more unified and understandable gene/allele name is recommended for ease of reading. For example, OgTPR1, TP, S1TPR, TPR, T, and S1-g were used to describe the same gene or genotype in the manuscript. This is not friendly to the readers. Reorganization of the names is recommended by referring to and observing genetic nomenclature for gene names and also allele designation. Please be very clear with regards to locus (consisting of three genes), genes and alleles, as well as their enumerating. Keep in mind that this may become a classical example in textbooks.
2. Line 68: " a lot of". So far the number is not a lot yet.
3. Line 76-77: "S1 is therefore the most important…". This claim seems to compare apple with pear.
4. Line 211: Allele 0 in genetics nomenclature in early days usually means a null allele.
5. Line 229: the result suggested that allele 1-4 was fixed in O. sativa. There seems to be an error here. Is it allele 1-3?
6. The writing does not read well. Need further editing and polishing.

Reviewer #3 (Remarks to the Author):

This manuscript reported genes responsible for hybrid sterility between Oryza sativa and Oryza

glaberrima. The authors found three closely linked genes, A4, TPR, and A6 in theS1 locus of O. glaberrima. The TPR encodes a protein containing two trypsin-like peptidase domains and a ribosome biogenesis regulatory domain, and it has been already published by the same group (Molecular Plant 2017). A6 encodes a peptidase protein with similar feature to the TPR peptidase, and it has been already reported by the same group and another group. A4 encodes an unknown protein and shown to interact with A6 and TPR using BiFc assay in this manuscript.
This manuscript is significant in the point reporting dual function of TPR: a complex of TPR, A4 and A6 makes up the killer system in sporophyte, and TPR alone has another role in the protection of TPR-containing gametes.

The hypothesis of the A4-TPR-A6 gamete killer-protector system is very complicated, and hard to understand for me at a glance, but careful reading of this manuscript and the previous paper published in Molecular Plant 2017 allowed me to understand that the working model in Fig. 5 is supported by the sufficient data employing transgenic japonica rice with TPR and CRISPR/Cas9 knockout mutants of NIL.

I have some comments for minor revisions.
(1) In Lines 78 and 79, some more descriptions are needed for OgTPR1 and SSP, including full name of the genes and what they encode. Most readers will not understand what they are until they read the previous papers.
(2) The names of some alleles in the text look wrong, based on the data of Supplementary Fig. 5. In line 229, "Alleles 1-4 and 1-8" should be Alleles 1-3 and 1-8; in line 239, "Alleles1-10 to 1-12" must be alleles 1-9 to 1-11; in line 240, "Alleles 1-13 and 1-14" should be 1-12 and 1-13; in line 241, "Alleles 1-15 and 1-16" must be alleles 1-14 and 1-15. Line 75 of Figure legend of Supplementary Figure 6 should be also corrected to "Alleles 1-3 and 1-8" from the current description of "Alleles 1-4 and 1-8"

Reviewer #4 (Remarks to the Author):

Review

This study dissects the genetic architecture of a major postzygotic reproductive barrier locus between two rice species, Oryza sativa and O. glaberrima. The motivation for this study is two-fold. First, reproductive barriers between rice taxa represent a major obstacle to breeding efforts. Second, detailed studies of reproductive barriers can shed new light on the evolution of species and are therefore of broad evolutionary interest.

In this study, the authors first use a combination of sophisticated experiments to characterize the S1 locus, a cluster of three genes that interact to confer reproductive isolation. Then, the authors characterize genetic variation at the S1 locus in the study species, as well as related species with the aim to reconstruct the evolutionary history of the locus.

The strength of this study is the detailed analysis of the molecular mechanism underlying HS mediated by the S1 locus, which provides interesting new insights into the genetic basis of HS in rice. Unfortunately, the study also suffers from major weaknesses. These include the clarity of the presentation, the conceptual basis motivating the different components of this study, and the evolutionary inferences.

1. Clarity of the presentation

This study is targeting a general audience and therefore must be understandable also to people who are not deeply into the field of HS in rice and into the details of analysing molecular gene functions

and gene interactions. In the present version, the manuscript is very difficult to follow.

For the analysis of the molecular mechanisms, information is necessary on why the chosen transgenic lines were produced and what expectations are for the different crosses performed with these lines. In part, this information is provided, but clearly not for all relevant lines / crosses. Please clarify which lines are hemi-, homo- or heterozygous and what the relevance of this is for your expectations and findings. Again, such information is partly provided but is missing in many instances.

Phenotypes are not well explained. Please provide more information on what organs show evidence for HS, pollen, spikelets, both? What about the female function (e.g. ovules)?

The term 'gametophytic manner' and 'sporophytic manner' appears to be colloquial language. Please use established terminology and provide an explanation upon first usage of these terms such that non-specialist readers can follow.

The legend to Figure 5b should be part of the main text and would provide relevant information to readers.

The extensive naming and renaming of genes (e.g. page 4) is confusing. Please use consistent naming for the genes throughout the manuscript and for example indicate in brackets alternative names for the same genes that have been used in other studies.

Some of the terms used to describe evolutionary processes and scenarios are not adequate and need to be revised. A case in point is the first sentence of the introduction: HS does not 'suppress gene migration' but (for example) 'reduces gene-flow' between populations or species. Evolution in general is not targeted or directional and therefore also HS does not have a purpose, i.e. 'to drive speciation and contribute to ...'.

Use of the term 'gene flows' in this manuscript is wrong and the term 'evolutionary flow' (page 8) does not exist. These parts need to be reformulated and appropriate terminology is essential. Otherwise, readers will not be able to follow the complex scenarios inferred.

2. Conceptual basis
The paper deals with the analysis of a killer-protector system. Please explain in the introduction what this is and / or refer to suitable recent review papers that provide adequate description.

The explanation provided of the Bateson-Dobzhansky-Muller incompatibility model is imprecise (i.e. 'certain speciation loci') and requires revision. First, it would be of help to be clear on the essence of the Bateson-Dobzhansky-Muller model. The standard description, with which most non-specialist readers may be most familiar, focuses on the origin and fixation of mutations at two (or more) loci in each of two lineages derived from a common ancestor that interact epistatically and have negative effects in the hybrids (e.g [1]). The locus investigated in the present study therefore does not reflect this standard definition because it consists of a single locus (S1) that is composed of three apparently highly linked genes that interact. This one-locus model also fits the Bateson-Dobzhansky-Muller model [2] but is not the standard model and therefore requires introduction.

It remains obscure how the Bateson-Dobzhansky-Muller incompatibility relates to meiotic drive and selfish genetic elements. Please clarify these aspects and / or drop reference to concepts that are not essential for the present study.

Use of the term 'speciation locus' is not warranted in the present study. Rarely, if ever, is speciation a result of very few or a single locus, and the role of the S1 locus in speciation has not been studied. S1 apparently plays an important role in current reproductive isolation between the two studied species but this tells us nothing about its potential role in the speciation process.

The motivation for trying to reconstruct the evolutionary history of the S1 locus is unclear. I presume that the authors tried to establish the point that the alleles evolved independently in two lineages, where they became fixed and cause HS when brought into contact. These points could be considered essential components of Bateson-Dobzhansky-Muller incompatibilities and may explain why so much emphasis has been put on the idea that alleles have become fixed.
To clarify this, a better explanation is required of what the key components of a Bateson-Dobzhansky-Muller incompatibility are in your view - and what scenarios / hypotheses you are testing.

3. Evolutionary inferences

The discussion of the evolution of the S1 locus is very difficult to follow and seems to mix up time-scales. The discussion about the role of Gondwanaland in the evolution of Asian and African rice, and O. meridionalis (page 10), is incompatible with dated divergence times between species in the Oryza genus [3] and the breakup of Gondwana. Briefly, Oryza originated in the Miocene (15 MYA), and Asia and African rice split in the Quaternary. The breakup of Gondwana occurred 200 MYA. These are vastly different timescales.

Unfortunately, foxtail millet appears far too diverged to be of much use in the present study. Data from more closely related taxa should be available and included in the analysis and discussion.

Based on the presented data on variation at the S1 locus within and among species, it appears that there is quite substantial variation within species and the common ancestor of O. sativa, glaberrima and meridionalis was most likely polymorphic at S1. Can the variation observed by a consequence of incomplete lineage sorting?

The present discussion entirely neglects the fact that Bateson-Dobzhansky-Muller incompatibilities can be polymorphic within species [1]. This aspect needs to be incorporated and discussed.

It remains unclear whether the 'S1 locus' behaves indeed as a single locus and why. Is there evidence for reduced recombination, maintaining allelic combinations (as evidenced potentially through elevated linkage disequilibrium (LD)) or are recombination rates similar to other genomic regions?

Cited references

1. Cutter, A.D., The polymorphic prelude to Bateson-Dobzhansky-Muller incompatibilities. Trends in Ecology & Evolution, 2012. 27(4): p. 209-218.
2. Sweigart, A.L. and J.H. Willis, Molecular evolution and genetics of postzygotic reproductive isolation in plants. F1000 Biol Rep, 2012. 4: p. 23.
3. Tang, L., et al., Phylogeny and biogeography of the rice tribe (Oryzeae): Evidence from combined analysis of 20 chloroplast fragments. Molecular Phylogenetics and Evolution, 2010. 54(1): p. 266-277.

Reviewers' comments:

Reviewer #1

This reviewer gave a positive overall comment and raised 4 major suggestions

Reviewer 1#: The manuscript dealt with 'An asymmetric allelic interaction drives allele transmission bias in interspecific rice hybrids". As well known, there is huge yield potential in the hybrid rice breeding. It is an important strategy for rice breeder to take advantage of this heterosis. However, hybrid sterility is a major form as reproductive isolation during domestication and hinders the exploitation of the heterosis displayed by inter-specific or inter-subspecific hybrid. In this manuscript, the authors demonstrated that three closely linked genes (*S1A4*, *S1TPR* and *S1A6*) in the African rice *S1* allele constitute a gamete killer-protector system. Knockout of anyone of the three genes at this locus can overcome the interspecific reproductive barrier. Evolutionary analysis showed that the *S1* loci arose from newly evolved genes and complex reorganization process. The manuscript would be of interest to a wide audience to understand the molecular mechanisms governing the incompatible interactions between the different species.

1. The cloned hybrid sterility loci, such as *S5*, *S7*, *Sa*, *Sc*, *S27*/*S28*, are closely adjacent genetic interaction. Why is the asymmetric genetic interaction localized at the *S1* locus? Whether do the interval genes, such as *A5* and so on, play a role in the genetic interaction?

Response: Two genetic models have been proposed for HS in rice. The HS loci *S5, S7, Sa,* and *Sc* fit the one-locus model, while *S27/S28, DPL1/DPL2,* and *DGS1/DGS2* fit the two-locus model. Moreover, emerging molecular evidence supports the conclusion that the loci that fit the one-locus HS model are usually complex loci, comprising multiple adjacent and functionally related genes. For example, we previously reported that the *Sa* locus contains the two adjacent genes *SaF* and *SaM*. In the *Sa* and *S5* systems, the symmetric allelic HS interactions are represented by the molecular interactions of the proteins from both alleles of the parental lines; for example, the interacting proteins in the *Sa* three-component complex, SaF^+ and SaM^+, are contributed by the *indica* allele,

while the SaM^{-} protein is from the *japonica* allele. Similarly, in the *S5* gamete-killer complex, the ORF5+ protein is encoded by the *indica* allele, but ORF4+ is encoded by the *japonica* allele. In the present study, the functional African rice allele *S1-g* consists of three closely linked active genes; *S1A4, S1TPR,* and *S1A6* (*SSP*). The gamete-killer function of *S1*-HS requires the three components (S1A4, S1TPR, and S1A6) only from *S1-g,* while the protector function depends on solely on *S1-g*-derived S1TPR; no component from the Asian rice allele is required for this gamete killer-protector system. The *S1* gamete killer-protector system is thus determined only by the African allele, representing an asymmetric allelic interaction. This character is distinct from the *S5* and *Sa* systems, which are symmetric allelic interactions involving components from both divergent alleles.

Our results showed that knockout of *S1A5* in the *S1-g* region did not affect pollen development in the NIL-*g* line, nor the hybrid sterility of the *S1* heterozygotes (Supplementary Fig. 3). On the other hand, the complementation of the *japonica* parental line RP-*s* with the three genes *S1A4, S1TPR,* and *S1A6* (without *S1A5* or the other putative ORFs from the *S1-g* region) could cause male and female sterility (Fig. 1 and Supplementary Fig. 5). We can therefore exclude the possibility that the other genes in this genetic interval are involved in *S1*-mediated HS.

2. The *S1* gene derived from African rice *Oryza glaberrima*, induced preferential abortion of both male and female gametes possessing its allelic alternative from Asian rice *O. sativa* only in the heterozygote. In this manuscript, the authors speculated that the co-existence of *A4*, *TPR* and *A6* might produce a sterility signal in a sporophytic manner to kill gametes (both male and female), moreover, *TPR* expressed in the microspores and *A6* (*SSP*) was not detected in EM and LM stage (Supplementary Fig. 1). It is better to provide more detailed data for expressions of the three genes in reproduction development?

Response: As suggested, we investigated the expression of the three genes in the anthers and panicles at various developmental stages using qRT-PCR. The new data are presented in Supplementary Fig. 2.

3. The subcellular localization of these three proteins A4, A6 and TPR were localized

in the nucleus, however, the authors showed that they produced a sterility signal in a sporophytic manner to kill gametes (both male and female). Is there any indirect result from these three gene interaction?

Response: We investigated the phenotypes of the single-gene knockout mutants *s1a4* and *s1a6*, revealing that six agronomically important traits, including their architecture, grain length, grain width, plant height, panicle length, and grain number per panicle, were not significantly affected; however, their 1000-grain weights were slightly increased, which may be related to the changes in amino acid metabolism hinted at by the transcriptome sequencing analysis. We therefore propose that the interactions of these three genes may have a pleiotropic effect on the seed-filling process in addition to their roles in HS. These results have been presented in Supplementary Fig. 4, and mentioned in the text (Page 5, lines 131–137).

4. It is better to discuss more about the mechanism? For example, whether does the detrimental sterility signal need to be emitted by a complex of the three genes? Is there any other possible mechanisms underlying the detrimental sterility signal?

Response: To explore the possible mechanisms involved, we conducted a transcriptome sequencing analysis. Six genes involved in the branched-chain amino acid (BCAA) degradation pathway showed significantly higher expression in the F_1 and NIL-*g* plants than in the RP-*s* plants (Supplementary Figs. 8–10). Dysfunctional BCAA biosynthesis is known to cause the abortion of male and female gametophyte development (Zhang *et al.*, 2015, *J. Exp. Bot.* 66: 879-888). The transcriptome data hinted that the BCAA deficiency may have a detrimental effect on gamete development; therefore, we propose that the complex of the three *S1-g* gene products may induce excessive BCAA degradation in F_1 and NIL-*g* plants, resulting in the sterility signal affecting gamete development. The S1TPR produced in *S1-g*-type gametes may maintain adequate levels of BCAA *via* its peptidase function, thereby rescuing the gametes from amino acid deficiency. The *S1-s*-type gametes in the F_1 hybrids lacking the functional S1TPR would still display a BCAA deficiency however, and fail to be restored to fertility. In Asian rice cultivars (*S1-sS1-s*), the genes involved in BCAA degradation are expressed at low levels, meaning that the gametes have enough BCAA to continue their development. We have added new data in Supplementary Figs. 8–

10 and mentioned the proposed mechanism in our revised manuscript (Pages 8–9, lines 238–254; Pages 13–14, lines 382–397).

Reviewer #2

This reviewer gave a positive overall comment and raised 4 major suggestions and 6 minor points

This paper identified a killer-protector system at *S1* locus responsible for hybrid sterility and segregation distortion between two cultivated rice species. This work substantially expanded our understanding of reproductive isolation. As shown by the work, the *S1* locus is an important speciation locus as it affects both male and female sterility in the hybrids. The data obtained from genetic analyses are sound and convincing, revealing a complex interaction among the three genes at this locus that form a killer-protector system in regulating hybrid sterility. Although killer-protector system has been reported previously, a novel and interesting feature is the characterization of one causal gene, TPR, that kills the gamete in hybrids at sporophytic stage and is also responsible for rescuing the gamete in a gameotophytic manner. The subsequent sequence and divergence analysis describes an evolutionary picture with respect to the origin of such reproductive barrier. Together with other reported studies, the results indicate that the killer-protector system may be a general strategy for postzygotic reproductive isolation during the evolution. In addition, this result may serve the first step for a feasible approach to overcome the inter-specific reproductive isolation to facilitate the utilization of distant heterosis in hybrid rice breeding.

1. The interaction of A4 with A6 and TPR is very critical to the proposed working model. However, genetic interaction does not necessarily mean physical interaction of the proteins. The evidence based on the present data for interaction is quite weak. And a further downside is that the work has provided no clue as to how and on what the killer works and the possible cellular and biochemical processes leading to gamete abortion. This left with a feeling that we came to an abrupt end. Some additional work seems necessary for smoother ending.

Response: We agree with the reviewer's comments. We have conducted

pull-down assays to confirm the interaction among the three proteins (Fig. 4c, 4d), the results of which indicated that S1A4 physically interacts with S1TPR and S1A6. These data are shown in Fig. 4c and 4d in the revised manuscript.

To probe the possible biochemical processes leading to gamete abortion, we performed a transcriptome sequencing analysis, revealing that the transcript levels of six genes involved in the BCAA degradation pathway were significantly higher in the F_1 and NIL-*g* plants than in RP-*s* (Supplementary Figs. 8–10). Dysfunctional BCAA biosynthesis is known to cause the abortion of male and female gametophyte development (Zhang *et al*., 2015, *J EXP BOT* 66: 879-888). Based on these observations, it is possible that the complex of the three proteins may induce excessive BCAA degradation, which would have a detrimental effect on fertility; therefore, we propose that the complex of the three *S1-g* gene products may induce excessive BCAA degradation in F_1 and NIL-*g* plants, resulting in the sterility signal affecting gamete development. The S1TPR produced in *S1-g*-type gametes may maintain adequate levels of BCAA *via* its peptidase function, thereby rescuing the gametes from amino acid deficiency. The *S1-s*-type gametes in the F_1 hybrids lacking the functional S1TPR would still display a BCAA deficiency and fail to be restored to fertility. In Asian rice cultivars (*S1-sS1-s*), the genes involved in BCAA degradation are expressed at low levels, meaning that the gametes have enough BCAA to continue their development. We have added new data in Supplementary Figs. 8–10 and mentioned these possible mechanisms in our revised manuscript (Pages 8–9, lines 238–254; Pages 13–14, lines 382–397).

2. As mentioned in the introduction, both male and female gametes carrying *S1-s* are selectively aborted. Thus how is the phenotype of the embryo-sac in the hybrid here?

Based on Fig. 2b, the author analyzed the segregation ratio based on the assumption that the female gametes with s/- were aborted. Is there any experiment to support the assumption? For Fig. 2a, I am wondering if ss/-- is not observed in the segregating population. If yes, to avoid ambiguity, ss/-- might be added in the genotype row with zero individual.

For Fig. 2c, the green, red, and blue colors are in accordance with the colors in Fig. 2b, which is quite clear. However, the white and grey colors have no correspondence in Fig, 2b, this might cause confusion.

Response: The histological defects in the development of embryo sacs of *S1* heterozygotes have been demonstrated in a number of previous studies (Koide *et al.*, 2008, *New Phytologist* 179: 888-900; Koide *et al.*, 2018, *Proc. Natl. Acad. Sci.* 115: E1955-E1962) To answer the reviewer's question about the phenotype of the embryo-sac, we conducted a histological observation of the embryo sacs of the hybrids derived from the crosses RP-*s* × NIL-*g* and *S1A4-S1A6*^{t} × *S1TPR*^{t}. The results were similar to those of the previous studies, indicating that gametes carrying *S1-s* or lacking the *S1TPR*^{t} transgene are selectively aborted during mega-gametogenesis (Supplementary Fig. 6).

Based on the genetic evidence presented in Fig. 2a, the co-segregations of the genotypes and phenotypes were consistent in the *S1*-heterozygotes carrying the transgenic *S1TPR*^{t} genotypes (*TT, T–, ––*), which strongly supports the notion that the female and male gametes containing *s*/– (*S1-s* without *S1TPR*^{t}) were aborted. No plants carrying the *ss*/–– genotype were observed in the segregating population; to avoid ambiguity, we accepted the reviewer's suggestion and added a new row for *ss*/–– containing no individuals.

We also appreciated the reviewer's suggestions regarding our figures. To make them clearer for readers, we added color codes in Fig. 2b according to the reviewer's suggestion. The color codes were also applied in Fig. 1f and Fig. 3d to avoid confusion.

3. I am wondering if the three components at *S1* have orthologs in other grass species. The Result said that one ortholog of TPR was identified in foxtail millet. If the orthologs existed in other species, a phylogenetic tree might tell something about the origin and relationship of these genes.

In addition, a haplotype network is recommended to trace the stepwise mutational step of different alleles. Is it possible that the most ancestral haplotype contains the A4-TPR-A6, and these genes were either lost or under rapid evolution in other linages?

It is not clear to me (or is it clear yet?), based on the presently available resequencing data from all sources, whether there are any *sativa* accessions that have all three genes (and/or other of the *A1* to *A6* genes) albeit that they may not be functional. This may have important implication in the evolutionary study.

Response: We thank the reviewer for this constructive suggestion. In

response to these comments, we used the nucleotide coding sequences of the three components of the *S1* locus as a query to perform a BLAST search of the *Poaceae*. The putative orthologs were used to construct a phylogenetic tree with the maximum likelihood method in MEGA7 (www.megasoftware.net/) and 1,000 bootstrap replications. We found that the nucleotide coding sequences of the *Oryza S1TPR* orthologs, but not *S1A4* and *S1A6*, were significantly similar to sequences in the genomes of *Zea mays*, *Sorghum bicolor*, *Brachypodium distachyon*, *Hordeum vulgare*, *Aegilops tauschii*, *Triticum aestivum*, and *Setaria italica* (Supplementary Fig. 11).

To determine the origin of the most ancestral *S1* haplotype, *O. officinalis* (CC genome species), *O. rhizomatis* (CC genome species), *O. eichingeri* (CC genome species), and *O. minuta* (BBCC genome species) were used as outgroups. The haplotypes of these species only harbor *S1TPR* and/or *S1TP* sequences, which may exclude the possibility of the haplotype carrying *S1A4-S1TPR-S1A6* being the most ancestral.

We previously aligned the genomic sequences of the *S1-g* and *S1-s* alleles with the publicly available sequences from the wild rice genome project and the 3000 Rice Genomes Project (https://registry.opendata.aws/3kricegenome/). This showed that only the *S1TP*-type haplotypes (with the premature stop codon), but not the *S1A4* and *S1A6* sequences, were present in all *O. sativa* and *O. rufipogon* accessions (Supplementary Figs. 12 and 13 and Supplementary Table 8).

4. Since you have no clue at the moment on what and how the killer works, it may be premature to state "this *S1* gamete killer-protector system is distinct from other Bateson-Dobzhansky-Muller type…". This is also related to the question in the previous paragraph about the evolutionary analysis.

Response: We have accepted the reviewer's suggestion, and clarified the difference of killer-protector system between *S1* locus and other loci in this revision (Page 13, lines 371–381).

Minor points:

1. Because of the complexity of the genes and alleles and interactions among the *S1* locus, a more unified and understandable gene/allele name is recommended for ease of reading. For example, OgTPR1, TP, S1TPR, TPR, T, and S1-g were used to

describe the same gene or genotype in the manuscript. This is not friendly to the readers. Reorganization of the names is recommended by referring to and observing genetic nomenclature for gene names and also allele designation. Please be very clear with regards to locus (consisting of three genes), genes and alleles, as well as their enumerating. Keep in mind that this may become a classical example in textbooks.

Response: In the revised version, we accepted the reviewer's suggestion and unified the different terms for the same gene or genotype in the main text of manuscript. *OgTPR1, S1TPR,* and *TPR* were unified as *S1TPR*; *S1A4* and *A4* were unified as *S1A4*; and *S1A6* and *A6* were unified as *S1A6*. Due to space limitations, the abbreviations *TPR, A4, A6, s,* and *g,* corresponding to *S1TPR, S1A4, S1A6, S1-s,* and *S1-g,* respectively, were still used in the figures, with definitions provided in the legends. To distinguish between the transgenes and the endogenous genes, we added superscript "t" after the name of transgenes.

2. Line 68: "a lot of". So far the number is not a lot yet.

Response: We changed "a lot of" to "several" (Page 3, line 77).

3. Line 76-77: "*S1* is therefore the most important…". This claim seems to compare apple with pear.

Response: We have deleted this description to tone down the claim in the revised manuscript (Page 3, line 87).

4. Line 211: Allele 0 in genetics nomenclature in early days usually means a null allele.

Response: We have changed the "allele 0" to "Allele 1" and amended all alleles with the correct numbers in the revised manuscript.

5. Line 229: the result suggested that allele 1-4 was fixed in *O. sativa*. There seems to be an error here. Is it allele 1-3?

Response: The reviewer is right. We apologize for our carelessness and have adjusted all alleles to reflect the amended order.

6. The writing does not read well. Need further editing and polishing.

Response: We have carefully revised our manuscript and asked a

professional editor to polish the writing quality in the current version.

Reviewer #3

This reviewer gave a positive overall comment and raised 2 minor points

This manuscript reported genes responsible for hybrid sterility between *Oryza sativa* and *Oryza glaberrima*. The authors found three closely linked genes, *A4*, *TPR*, and *A6* in the *S1* locus of *O. glaberrima*. The *TPR* encodes a protein containing two trypsin-like peptidase domains and a ribosome biogenesis regulatory domain, and it has been already published by the same group (Molecular Plant 2017). A6 encodes a peptidase protein with similar feature to the TPR peptidase, and it has been already reported by the same group and another group. *A4* encodes an unknown protein and shown to interact with A6 and TPR using BiFC assay in this manuscript.

This manuscript is significant in the point reporting dual function of TPR: a complex of TPR, A4 and A6 makes up the killer system in sporophyte, and TPR alone has another role in the protection of TPR-containing gametes.

The hypothesis of the A4-TPR-A6 gamete killer-protector system is very complicated, and hard to understand for me at a glance, but careful reading of this manuscript and the previous paper published in Molecular Plant 2017 allowed me to understand that the working model in Fig. 5 is supported by the sufficient data employing transgenic *japonica* rice with TPR and CRISPR/Cas9 knockout mutants of NIL.

I have some comments for minor revisions.

1. In Lines 78 and 79, some more descriptions are needed for *OgTPR1* and *SSP*, including full name of the genes and what they encode. Most readers will not understand what they are until they read the previous papers.

Response: We provided this detailed information in the revised manuscript (Page 3, lines 88–89).

2. The names of some alleles in the text look wrong, based on the data of Supplementary Fig. 5. In line 229, “Alleles 1-4 and 1-8” should be Alleles 1-3 and 1-8; in line 239, “Alleles1-10 to 1-12” must be alleles 1-9 to 1-11; in line 240, “Alleles 1-13 and 1-14” should be 1-12 and 1-13; in line 241, “Alleles 1-15 and 1-16” must be

alleles 1-14 and 1-15. Line 75 of Figure legend of Supplementary Fig. 6 should be also corrected to "Alleles 1-3 and 1-8" from the current description of "Alleles 1-4 and 1-8"

Response: The reviewer is right. We apologize for our carelessness and thank the reviewer for their observation. In current revision, we have amended all alleles with the correct numbers.

Reviewer #4

This reviewer gave a positive overall comment and raised 14 suggestions involved in 3 specific aspects

This study dissects the genetic architecture of a major postzygotic reproductive barrier locus between two rice species, *Oryza sativa* and *O. glaberrima*. The motivation for this study is two-fold. First, reproductive barriers between rice taxa represent a major obstacle to breeding efforts. Second, detailed studies of reproductive barriers can shed new light on the evolution of species and are therefore of broad evolutionary interest. In this study, the authors first use a combination of sophisticated experiments to characterize the *S1* locus, a cluster of three genes that interact to confer reproductive isolation. Then, the authors characterize genetic variation at the *S1* locus in the study species, as well as related species with the aim to reconstruct the evolutionary history of the locus. The strength of this study is the detailed analysis of the molecular mechanism underlying HS mediated by the *S1* locus, which provides interesting new insights into the genetic basis of HS in rice.

Unfortunately, the study also suffers from major weaknesses. These include the clarity of the presentation, the conceptual basis motivating the different components of this study, and the evolutionary inferences.

1. Clarity of the presentation

1.1 This study is targeting a general audience and therefore must be understandable also to people who are not deeply into the field of HS in rice and into the details of analysing molecular gene functions and gene interactions. In the present version, the manuscript is very difficult to follow.

Response: We have carefully revised our manuscript according to the

specific suggestions raised by all reviewers, making it more understandable for general audiences. We also asked a professional editor to polish the current version.

1.2 For the analysis of the molecular mechanisms, information is necessary on why the chosen transgenic lines were produced and what expectations are for the different crosses performed with these lines. In part, this information is provided, but clearly not for all relevant lines / crosses. Please clarify which lines are hemi-, homo- or heterozygous and what the relevance of this is for your expectations and findings. Again, such information is partly provided but is missing in many instances.

Response: We have supplied this detailed information in the revised manuscript. Please refer to Page 5, lines 122–124, lines 143–145; Page 7, lines 184–188; and Page 8, lines 210–213.

1.3 Phenotypes are not well explained. Please provide more information on what organs show evidence for HS, pollen, spikelets, both? What about the female function (e.g. ovules)?

Response: We mainly explored the HS phenotype of the pollen and spikelets. Several studies have reported histological defects in the development of pollen grains and embryo sacs in *S1* heterozygotes (Koide *et al.*, 2008, *New Phytologist* 179: 888-900 and Koide *et al.*, 2018, *Proc. Natl. Acad. Sci.* 115: E1955-E1962). To answer the reviewer's question about the female function, we also conducted a histological observation of the embryo sacs of hybrid plants derived from RP-*s* × NIL-*g* and *S1A4-S1A6*^{t} × *S1TPR*^{t}. Our results were similar to those of previous studies, indicating that the gametes carrying *S1-s* or lacking *S1TPR*^{t} are selectively aborted during mega-gametogenesis (Supplementary Fig. 6).

1.4 The term 'gametophytic manner' and 'sporophytic manner' appears to be colloquial language. Please use established terminology and provide an explanation upon first usage of these terms such that non-specialist readers can follow.

Response: In the field of plant reproductive biology, "gametophytic manner" and "sporophytic manner" are standard terms used to indicate that the genetic manner or phenotype is determined by the gametophytic genotype or the sporophytic genotype, respectively (Yu *et al.*, 2018, *Science* 360: 1130-1132;

Chhun *et al.*, 2007, *Plant Cell* 19: 3876–3888; Itabashi *et al.*, 2011, *Plant J* 65: 359-367). To make these terms more accessible to non-specialist readers, we accepted the reviewer's suggestion, and made the description of gametophytic and sporophytic effects a bit simpler for the general reader (Page 2, lines 37-39; Page 6, lines 163–164, lines 168–172, line 175).

1.5 The legend to Figure 5b should be part of the main text and would provide relevant information to readers.

Response: We accepted the reviewer's suggestion and mentioned Fig. 5b in the main text (Page 12, Lines 344–355).

1.6 The extensive naming and renaming of genes (e.g. page 4) is confusing. Please use consistent naming for the genes throughout the manuscript and for example indicate in brackets alternative names for the same genes that have been used in other studies.

Response: We have accepted the reviewer's suggestion and unified the names of the genes. *OgTPR1*, *S1TPR*, and *TPR* were unified as *S1TPR*; *S1A4* and *A4* were unified as *S1A4*; and *S1A6* and *A6* were unified as *S1A6* in the revised manuscript. Due to space limitations, abbreviations for the genes and alleles are still used in the figures, with definitions provided in the legends.

1.7 Some of the terms used to describe evolutionary processes and scenarios are not adequate and need to be revised. A case in point is the first sentence of the introduction: HS does not 'suppress gene migration' but (for example) 'reduces gene-flow' between populations or species. Evolution in general is not targeted or directional and therefore also HS does not have a purpose, i.e. 'to drive speciation and contribute to ...'. Use of the term 'gene flows' in this manuscript is wrong and the term 'evolutionary flow' (page 8) does not exist. These parts need to be reformulated and appropriate terminology is essential. Otherwise, readers will not be able to follow the complex scenarios inferred.

Response: We thank the reviewer for sharing their expertise in evolutionary biology and corrected these terms according to the reviewer's suggestion. We rewrote this section of the text using the appropriate terms (Page 2, lines 51–52; Page 11, lines 306 and 310).

2. Conceptual basis

2.1 The paper deals with the analysis of a killer-protector system. Please explain in the introduction what this is and / or refer to suitable recent review papers that provide adequate description.

Response: As suggested, we added a brief description of the killer-protector system and cited a recent review paper (Bravo *et al.*, 2018, *Trends Genet.*, 34: 424-433) in the Introduction section (Page 3, lines 60–67).

2.2 The explanation provided of the Bateson-Dobzhansky-Muller incompatibility model is imprecise (i.e. 'certain speciation loci') and requires revision. First, it would be of help to be clear on the essence of the Bateson-Dobzhansky-Muller model. The standard description, with which most non-specialist readers may be most familiar, focuses on the origin and fixation of mutations at two (or more) loci in each of two lineages derived from a common ancestor that interact epistatically and have negative effects in the hybrids (e.g [1]). The locus investigated in the present study therefore does not reflect this standard definition because it consists of a single locus (*S1*) that is composed of three apparently highly linked genes that interact. This one-locus model also fits the Bateson-Dobzhansky-Muller model [2] but is not the standard model and therefore requires introduction.

It remains obscure how the Bateson-Dobzhansky-Muller incompatibility relates to meiotic drive and selfish genetic elements. Please clarify these aspects and / or drop reference to concepts that are not essential for the present study.

Response: We thank the reviewer for this comment. We have now cited these references and reorganized this part of the introduction.

Meiotic drivers, also called 'ultra-selfish' genes, are preferentially transmitted into more than half (sometimes all) of the meiotic products and progeny of heterozygotes by destroying the other viable meiotic products lacking the driver, leading to segregation distortion (Bravo *et al.*, 2018, *Trends Genet.* 34: 424-433). Similarly, the HS loci act as selfish genetic elements that eliminate competing alleles to gain a transmission advantage. Both result in Bateson-Dobzhansky-Muller incompatibility. We clarified these aspects in the Introduction (Page 2–3, lines 52–60).

2.3 Use of the term 'speciation locus' is not warranted in the present study. Rarely, if ever, is speciation a result of very few or a single locus, and the role of the *S1* locus in speciation has not been studied. *S1* apparently plays an important role in current reproductive isolation between the two studied species but this tells us nothing about its potential role in the speciation process.

Response: We changed ‘speciation locus’ to ‘HS locus’ in the current version.

2.4 The motivation for trying to reconstruct the evolutionary history of the *S1* locus is unclear. I presume that the authors tried to establish the point that the alleles evolved independently in two lineages, where they became fixed and cause HS when brought into contact. These points could be considered essential components of Bateson-Dobzhansky-Muller incompatibilities and may explain why so much emphasis has been put on the idea that alleles have become fixed. To clarify this, a better explanation is required of what the key components of a Bateson-Dobzhansky-Muller incompatibility are in your view - and what scenarios / hypotheses you are testing.

Response: The reviewer is correct, we did want to find the point that the alleles evolved independently in two lineages. We stated this motivation in the text to make this clearer for readers (refer to Page 9, lines 258–264; Page 10, lines 271-277, lines 282–286).

3. Evolutionary inferences

3.1 The discussion of the evolution of the *S1* locus is very difficult to follow and seems to mix up time-scales. The discussion about the role of Gondwanaland in the evolution of Asian and African rice, and *O. meridionalis* (page 10), is incompatible with dated divergence times between species in the *Oryza* genus [3] and the breakup of Gondwana. Briefly, *Oryza* originated in the Miocene (15 MYA), and Asia and African rice split in the Quaternary. The breakup of Gondwana occurred 200 MYA. These are vastly different timescales.

Response: The extensive geographic distribution of *Oryza* species is a longstanding question, which has been explained by the Gondwanaland origin theory (Chang *et al.*, 1976, *Euphytica*, 25, 425-441; Khush *et al.*, 1997, *Plant Mol. Biol.*, 35, 25-34; Wambugu *et al.*, 2015, *Sci. Rep.* 5, 13957). We agree that the current divergence times based on the molecular evolution between species in the

***Oryza* genus are not in accordance with a Gondwanaland origin (Tang *et al.* 2010, *Mol. Phylogenet. Evol.* 54, 266-277; Stein *et al.*, 2018, *Nat. Genet.* 50, 285-296), however, even molecular evolutionary biologists have been unable to determine the exact divergence times of the *Oryza* species, largely because of the incomplete lineage sorting of ancestral polymorphisms (Stein *et al.*, 2018, *Nat. Genet.* 50, 285-296). The deduced divergence times of the *Oryza* genus are based on a sequence analysis of the currently available species, which may cause artifacts or bias in the algorithm due to the absence of extinct ancestral species. We have therefore toned down but kept our deduction that the *S1* locus of *O. rufipogon*/*O. sativa* and *O. bathii*/*O. glaberrima* might have originated in ancestral species in Gondwanaland, diverging as the continents separated (refer to Page 14, lines 398–407). In addition, we added the possibility that *O. sativa* and *O. glaberrima* might have been derived from the incomplete lineage sorting of ancestral polymorphisms at the *S1* locus (refer to Pages 14-15, lines 420–432).**

3.2 Unfortunately, foxtail millet appears far too diverged to be of much use in the present study. Data from more closely related taxa should be available and included in the analysis and discussion.

Based on the presented data on variation at the *S1* locus within and among species, it appears that there is quite substantial variation within species and the common ancestor of *O. sativa*, *glaberrima* and *meridionalis* was most likely polymorphic at *S1*. Can the variation observed by a consequence of incomplete lineage sorting? The present discussion entirely neglects the fact that Bateson-Dobzhansky-Muller incompatibilities can be polymorphic within species [1]. This aspect needs to be incorporated and discussed.

Response: We used the nucleotide sequences of the three components of the *S1* locus as templates to perform a BLAST search of the *Poaceae*. We found that the putative ortholog of *S1TPR* in *Setaria italica* is similar to *S1TPR* in African rice (Supplementary Fig. 11). In response to the reviewer's suggestion, we further tested the accessions of closely related species in *Oryza* as outgroups, including *O. officinalis* (CC genome species), *O. rhizomatis* (CC genome species), *O. eichingeri* (CC genome species), and *O. minuta* (BBCC genome species) (Supplementary Figs. 12 and 13 and Supplementary Table 8). We found that the *S1A4* and *S1A6* orthologs are not present in these species, although all of them had putative

***S1TPR* orthologs containing C at SNP site 7, corresponding to the premature stop codon in S1TP. These results suggest that *S1A4* and *S1A6* probably arose from newly evolved genes in the AA-genome *Oryza* species, and that variants of this site 7 SNP in *S1TPR* and *S1TP* co-existed in the primitive *Oryza* gene pool (Supplementary Figs. 12 and 13 and Supplementary Table 8). We analyzed and described these new data in our revised manuscript (Page 10, lines 271–295).**

We agree with the reviewer that the extensive variations in the *S1* locus observed in the ancestral *Oryza* species may have been polymorphic in the common ancestor. Our new results indicate that ancestral polymorphisms were likely to have existed (Supplementary Figs. 12 and 13 and Supplementary Table 8). Despite the clear outline of the *Oryza* phylogeny, the exact relationships among the *Oryza* species is elusive, resulting from gene tree discordances caused by factors such as incomplete lineage sorting (Stein, *et al.*, 2018, *Nat. Genet.* 50: 285-296). Based on these data, we included the possibility that the variations might be a consequence of incomplete lineage sorting and discussed this in the revised manuscript (Pages 14–15, lines 420–432).

3.3 It remains unclear whether the '*S1* locus' behaves indeed as a single locus and why. Is there evidence for reduced recombination, maintaining allelic combinations (as evidenced potentially through elevated linkage disequilibrium (LD)) or are recombination rates similar to other genomic regions?

Response: The *S1* locus has repeatedly been shown to function as a single locus (Sano, 1990, *Genetics* 125: 183-191; Koide *et al.*, 2008, *New Phytologist* 179: 888-900; Koide *et al.*, 2018, *Proc. Natl. Acad. Sci.* 115: E1955-E1962; Garavito *et al.*, 2010, *Genetics* 185: 1425-1440; Xie *et al.*, 2017, *Mol. Plant* 10: 1137-1140). We have mentioned these publications in the revised manuscript (Page 3, lines 83-84). In our previous study, 11,000 individuals were used to finely map *S1* to a 29-kb region (at *S1-g*), which contains these three *S1*-related genes. Among these individuals, only two recombinants with recombination sites within this 29-kb region were detected in the *S1* mapping region (Xie *et al.*, 2017, *Mol. Plant* 10: 1137-1140). The sequence structures between *S1-g* and *S1-s* are so different (see Figure 1a) that the meiotic pairing and recombination between the *S1-g* and *S1-s* intervals should be largely suppressed, making this region behave as a single complex locus.

Reviewer #1 (Remarks to the Author):

The manuscript NCOMMS-18-34859A dealt with “An asymmetric allelic interaction drives allele transmission bias in interspecific rice hybrids”. In this revision, the authors present a significantly improved version of their manuscript. In my opinion, they have correctly addressed most of the points raised in my original review. They investigated the expression of the three genes in the anthers and panicles at various developmental stages, conducted a transcriptome sequencing analysis, and found that six genes involved in the branched-chain amino acid degradation pathway might have a detrimental effect on gamete development. Based on these results, the authors showed that three closely linked genes (S1A4, S1TPR and S1A6) in the African S1 allele constitute a killer and protector system that eliminates gametes carrying the Asian allele, but S1TPR can rescue S1-g gametes. S1TPR is a killer and also is a protector that is distinguished from the reported kill-protector system. Better to add this in Discussion.

Reviewer #2 (Remarks to the Author):

The revised manuscript has well addressed the questions of reviewer 2 and is suitable for acceptance. One additional minor point: line 77-80, a recently cloned locus ESA1 for interspecific hybrid incompatibility might be added to the introduction.

The major concerns of reviewer 4 involved three aspects of the previous manuscript including presentation, explanation of the models, and evolutionary analysis.
Because of the complexity of interactions in S1 (which involved a number of transgenes and hybridizations in functional characterization), reviewer 4 asked for a clearer elaboration of the genetic materials and genetic design of the experiments (as well as the expectations, phenotypes and several concepts). Now the revised version is much easier to read and understand.
Reviewer 4 also emphasized the interpretation of genetic models (BDM model and killer-protector model). These models are well explained in the background and touched in the results in the revised manuscript.
Finally, the evolutionary analyses have been improved in the revised version. More convinced evidences are given for inference of the origin and divergence of S1 locus.
Taken together, the comments have been appropriately addressed.

Reviewer #3 (Remarks to the Author):

This is a revised manuscript that I have reviewed previously.
The authors revised it incorporating my comments. According to the comments and suggestions by other reviewers, they have also included new data such as expression analysis of the candidate genes in anthers and panicles at different developmental stage, transcriptome analysis of anthers, pull-down assay demonstrating the interaction among the candidate proteins, and discussion of mechanism. This manuscript has been revised satisfactorily.

Reviewer #4: unavailable.

REVIEWERS' COMMENTS:

Reviewer #1 (Remarks to the Author):

The manuscript NCOMMS-18-34859A dealt with “An asymmetric allelic interaction drives allele transmission bias in interspecific rice hybrids”. In this revision, the authors present a significantly improved version of their manuscript. In my opinion, they have correctly addressed most of the points raised in my original review. They investigated the expression of the three genes in the anthers and panicles at various developmental stages , conducted a transcriptome sequencing analysis , and found that six genes involved in the branched-chain amino acid degradation pathway might have a detrimental effect on gamete development. Based on these results, the authors showed that three closely linked genes (S1A4, S1TPR and S1A6) in the African S1 allele constitute a killer and protector system that eliminates gametes carrying the Asian allele, but S1TPR can rescue S1-g gametes. S1TPR is a killer and also is a protector that is distinguished from the reported kill-protector system. Better to add this in Discussion.

Response: We thank the reviewer for positive comments and have added the dual function of S1TPR in Discussion (refer to Page 13, Lines 374-377).

Reviewer #2 (Remarks to the Author):

The revised manuscript has well addressed the questions of reviewer 2 and is suitable for acceptance. One additional minor point: line 77-80, a recently cloned locus ESA1 for interspecific hybrid incompatibility might be added to the introduction.

The major concerns of reviewer 4 involved three aspects of the previous manuscript including presentation, explanation of the models, and evolutionary analysis.

Because of the complexity of interactions in S1 (which involved a number of transgenes and hybridizations in functional characterization), reviewer 4 asked for a clearer elaboration of the genetic materials and genetic design of the experiments (as well as the expectations, phenotypes and several concepts). Now the revised version is much easier to read and understand.

Reviewer 4 also emphasized the interpretation of genetic models (BDM model and killer-protector model). These models are well explained in the background and touched in the results in the revised manuscript.

Finally, the evolutionary analyses have been improved in the revised version. More convinced evidences are given for inference of the origin and divergence of S1 locus.

Taken together, the comments have been appropriately addressed.

Response: We thank the reviewer for positive comments. As requested, we cited a recent reference and added this information in the Introduction section (refer to Page3, Lines 76-77).

Reviewer #3 (Remarks to the Author):

This is a revised manuscript that I have reviewed previously.

The authors revised it incorporating my comments. According to the comments and suggestions by other reviewers, they have also included new data such as expression analysis of the candidate genes in anthers and panicles at different developmental stage, transcriptome analysis of anthers, pull-down assay demonstrating the interaction among the candidate proteins, and discussion of mechanism. This manuscript has been revised satisfactorily.

Response: We thank the reviewer for positive comments.

Reviewer #4: unavailable.

Response: We thank the reviewer 2 for evaluating the comments raised by reviewer #4.
